# Generation of iPSCs carrying a common LRRK2 risk allele for *in vitro* modeling of idiopathic Parkinson's disease

**DOI:** 10.1371/journal.pone.0192497

**Published:** 2018-03-07

**Authors:** Lara Marrone, Christine Bus, David Schöndorf, Julia Catherine Fitzgerald, Manuela Kübler, Benjamin Schmid, Peter Reinhardt, Lydia Reinhardt, Michela Deleidi, Tanya Levin, Andrea Meixner, Barbara Klink, Michael Glatza, Christian Johannes Gloeckner, Thomas Gasser, Jared Sterneckert

**Affiliations:** 1 DFG-Center for Regenerative Therapies Technische Universität Dresden (CRTD), Dresden, Germany; 2 Department of Neurodegenerative Diseases, Centre of Neurology and Hertie-Institute for Clinical Brain Research, University of Tübingen, Tübingen, Germany; 3 German Center for Neurodegenerative Diseases (DZNE), Tübingen, Germany; 4 Max Planck Institute for Molecular Biomedicine, Münster, Germany; 5 Institut für Klinische Genetik, Medizinische Fakultät Carl Gustav Carus, Technische Universität Dresden, Dresden, Germany; 6 Centre for Ophthalmology, Institute for Ophthalmic Research, University of Tübingen, Tübingen, Germany; Universita degli Studi di Padova, ITALY

## Abstract

Induced pluripotent stem cells (iPSCs) have recapitulated several aspects of Parkinson’s disease (PD), but most iPSCs are derived from familial cases, which account for only about 15% of patients. Thus, while the emphasis has justifiably been on using iPSCs to model rare familial cases, models for the most common forms of PD are critically lacking. Here, we report the generation of an iPSC-based model of idiopathic PD (iPD) with or without RS1491923, which is a common risk variant in the *LRRK2* locus. Consistent with GWA studies, we found large variability in our datasets. However, iPSC-derived neurons carrying the risk allele emerged for displaying subtle disturbances of cellular degradative systems, in line with familial PD models. We also observed that treatment with the LRRK2 inhibitor CZC-25146 slightly reduced a marker of aSYN pathology in all iPD lines. Future iPSC-based studies may need to be structured similarly to large GWA studies in order to obtain relevant statistical power. However, results from this pilot study suggest that iPSC-based modeling represents an attractive way to investigate idiopathic diseases.

## Introduction

Parkinson’s disease (PD) is a neurodegenerative disease characterized by the loss of midbrain dopaminergic neurons (mDANs) in the *substantia nigra pars compacta*, which results in bradykinesia, tremor, muscular rigidity, postural instability, and, in some cases, cognitive impairment. PD ranks as the second most common neurodegenerative disease after Alzheimer’s disease [1], and patient specific induced pluripotent stem cells (iPSCs) have shown great promise in recapitulating critical aspects of PD pathogenesis, enabling mechanistic studies as well as the identification of novel therapeutics [2]. However, virtually all iPSC-based models are derived from familial cases (fPD), which account for only about 10–15% of PD cases. The remaining 85–90% are idiopathic (iPD). The total heritability of PD has been estimated at about 30%, the majority of which may be mediated through common genetic risk alleles [3]. Therefore, results from fPD models might only partially apply to iPD. Thus, it is critical to develop models of iPD, and iPSCs are one of the only methods available to investigate sporadic diseases using human neurons.

*LRRK2* G2019S is the most common mutation known to cause fPD [4], and patients with LRRK2 G2019S exhibit comparable symptoms and disease progression to iPD cases [5, 6], therefore understanding the interplay between LRRK2 activity and iPD pathogenesis is of great interest. LRRK2 is a large multi-domain protein whose exact physiological functions are still under debate. Its two distinct enzymatic domains, the ROC-GTPase domain and the kinase domain, would catalyze GTP-GDP hydrolysis and phosphorylation of several substrates, respectively. It is suggested that LRRK2, along with TAU and alpha-synuclein (aSYN) [7], two other proteins central to PD, interact to trigger PD pathology in at least a subset of iPD patients. At least two lines of evidence indicate that LRRK2 is involved in iPD pathogenesis. First, patient genotyping has shown that G2019S, which causes fPD, is present in some cases of iPD, suggesting that LRRK2 might play a role in a subset of iPD patients [8, 9]. However, these cases are relatively rare. Genome-wide association (GWA) studies have contributed a second line of evidence by showing that common variations around *LRRK2* modulate the risk of acquiring iPD [10, 11]. For example, the single nucleotide polymorphisms (SNP) RS1491923 was shown to have an odds ratio (OR) of 1.14 (P = 10^−5^) [10].

Because patient neurons are inaccessible and animal models do not develop many of the pertinent features of PD, induced pluripotent stem cells (iPSCs) are promising tools for modeling PD. iPSCs can proliferate without limit while maintaining their potential to generate derivatives of all germ layers, including mDANs. Additionally, they can be generated from cells from patients with observable disease phenotypes and known, or even unknown, genotypes. Previously, our group and others showed that iPSCs from PD patients with *LRRK2* G2019S can recapitulate hallmarks of PD pathogenesis [12–16]. Using neurons differentiated from these iPSCs, we demonstrated that *LRRK2* G2019S induced increased aSYN and TAU levels, caused aberrant mitochondrial function and trafficking, increased ERK phosphorylation, decreased neurite outgrowth, disrupted autophagy, and increased dopaminergic neurodegeneration. Because PD is multifactorial and LRRK2 phenotypes are modified by polymorphisms in the genetic background [17, 18], our lab previously generated isogenic gene-corrected iPSCs. Importantly, we demonstrated that PD phenotypes were only robustly detected when comparing neurons with *LRRK2* G2019S to isogenic gene corrected controls [12].

Since G2019S increases LRRK2 kinase activity, brain penetrant small molecules specifically inhibiting LRRK2 are being developed as possible treatments for PD. Experiments *in vitro* and *in vivo*, including using iPSC-derived models, suggest that inhibition of LRRK2 ameliorates PD pathogenesis associated with G2019S [12, 15, 19, 20]. However, it is unclear whether inhibition of LRRK2 would be beneficial for idiopathic PD patients lacking coding sequence mutations in LRRK2. In this context, iPSCs generated from iPD samples with different risk alleles would be a potentially powerful approach to assessing the contribution of LRRK2 to iPD as well as to evaluate LRRK2 inhibition in different PD subtypes.

Here, we report the generation of the first iPSC-model of iPD, either with or without a common risk variant at the LRRK2 locus. Because SNPs at the SNCA and MAPT locus are the strongest common risk variants for PD, we made sure that all lines were identical with respect to the variants SNCA (G/G, rs356219) and MAPT (A/A, rs393152). iPSCs were generated from four iPD patients. Of these, two patients carried the LRRK2-RS1491923 risk allele C/C, and two carried the non-risk T/T variant. Of note, while RS1491923 tags the haplotype that is associated with PD risk, the actual functional risk variant remains unknown. Therefore, it is not possible to generate isogenic iPSCs, but age and gender matched healthy controls were included for comparison. In addition, because LRRK2-RS1491923 does not cause fPD, we expected phenotypes to be subtle. Indeed, phenotype detection proved rather cumbersome, but consistent with familial PD models pathogenesis [12–16], neurons with C/C-risk allele exhibited slight differences in autophagy and mitochondrial protein clearance compared to healthy controls. To test if LRRK2 inhibition could rescue PD phenotypes, we first evaluated target engagement by the small molecule CZC-25146, a known and highly regarded inhibitor of LRRK2. Phosphorylation of LRRK2 serines 910 and 935 are among the validated biomarkers of LRRK2 target engagement. Because LRRK2 protein levels are very low in mDANs, we used gene targeting to introduce a tandem-affinity tag, which was used to validate effective inhibition of LRRK2 by CZC-25146 on purified LRRK2 protein. Inhibition of LRRK2 in our iPSC-derived mDANs slightly reduced aSYN levels specifically in iPD samples and not in healthy controls, where aSYN remained relatively low. Although our data did not reach full statistical significance due to the low number of lines involved in this pilot study, this work suggests the utility of follow-up studies based on these findings. As learnt from GWA studies, an important aspect of future work will involve incorporating a higher number of patient-derived cell lines to increase statistical significance and facilitate the identification of correlations between genotypes and phenotypes, particularly to aim for genotype stratification and generation of subtype-specific *in vitro* models for drug testing, a critical component of personalized medicine.

## Materials and methods

### Ethics statement

Informed consent was obtained for all patients and healthy individuals who donated samples for this study. Consent was obtained using a written protocol previously approved by the instutional review board Ethik-Kommission der Medizinischen Fakultät am Universitätsklinikum Tübingen. *In vitro* experiments were carried out with cell lines obtained and established from the above mentioned individuals unless differently stated.

### iPSC generation and characterization

With the exception of the iPSC line Control 3 [21], all iPSCs lines used in this study were newly generated. Dermal fibroblasts obtained from skin biopsies of patients with iPD and healthy controls were initially cultured in fibroblast medium, which consisted of Knock-out DMEM supplemented with 10% fetal calf serum, 1% penicillin/streptomycin (Merck Millipore)/glutamine (PSG), and 1% nonessential amino acids (both Biochrom). Reprogramming was performed using either the CytoTune-iPS 2.0 Sendai Reprogramming kit (Thermo Fisher Scientific) or episomal plasmids. Sendai virus-mediated reprogramming was performed according to manufacturer instructions as follows: fibroblasts at a passage of 6 or lower were seeded into at least two wells of a 6-well plate. One well was used to perform a cell count and calculate the virus volume for transduction.

$$Volume\;of\;virus\;(\mu l)=\frac{MOI\;(\frac{CIU}{cell})\;x\;number\;of\;cells}{virus\;titer\;(\frac{CIU}{ml})\;x\;10^{-3}\;(\mu \frac{l}{ml})}$$

KOS MOI = 5, c-MYC MOI = 5, Klf4 MOI = 3

Fibroblasts were transduced when 70% confluent. On the day after, cells were washed 5x with fresh complete fibroblast medium to remove the reprogramming vectors. In the second approach, fibroblasts were nucleofected with episomal plasmids containing hOCT4, hSOX2 and hKLF4, and hL-MYC and hLIN28 (pCXLE-hOCT3/4, pCXLE-hSK and pCXLE-hUL (AddGene)) [22] using an NHDF nucleofection kit (Lonza) and the Nucleofector® II (Amaxa Biosystems). After a few days, transduced or transfected cells were seeded on Matrigel- (Corning) coated 10 cm cell culture dishes at a confluence of 2x10^5^ cells. Medium was changed to Essential 6 Medium (Gibco), and replaced every day from then on for one week, when medium was switched to TeSR-E8 (STEM CELL Technologies). Approximately three weeks after, putative iPSC colonies had grown to an appropriate size for transfer and were manually picked and further expanded to establish lines. Pluripotency was assessed by immunofluorescent staining and qRT-PCR. Additionally, for the identification of plasmid integration-free clones, DNA was isolated using the DNeasy® Blood and Tissue Kit (Qiagen). Primers specific for the exogenes (OCT3/4-exo_FW: CAT TCA AAC TGA GGT AAG GG, OCT3/4-exo_RV: TAG CGT AAA AGG AGC AAC ATA, SOX2-exo_FW: TTC ACA TGT CCC AGC ACT ACC AGA, SOX2-exo_RV: TTT GTT TGA CAG GAG CGA CAA T, L-MYC-exo_FW: GGC TGA GAA GAG GAT GGC TAC, L-MYC-exo_RV: TTT GTT TGA CAG GAG CGA CAA T) were used to determine integration-free clones by PCR. Plasmid DNA from pCXLE-hOCT3/4, pCXLE-hSK and pCXLE-hUL (addgene) were used as positive controls. Eventually, cell lines were also karyotyped to ensure euploidy.

### Karyotyping

Cytogenetics was performed by either conventional G-banding or SNP array. For metaphase spread, cells were treated with colcemid when 50% confluent (0.35 μg/ml for 4 h), incubated in 75 mM KCl for 20 min at 37°C, and fixed in freshly prepared methanol/acetic acid (3:1) at room temperature. The cell suspension was dropped onto glass slides and G-banding was performed using standard protocols. SNP genotyping was performed on whole cell genome using the Infinium OmniExpressExome-8 BeadChip (Illumina). The data was analysed with Illumina BeadStudio.

### Generation of LRRK2 SF-TAP tagged iPSC lines

TALENs targeting LRRK2 exon 1 were designed as described by Cermak et al (Nucleic Acids Res, 2011); TALEN1: AGTCAGGCTGAACAAT, TALEN2: AGATAGAAACGCTGGTCCA. The LRRK2 SF-TAP TAG donor construct was generated from multiple PCR amplifications as shown in S4 Fig. Primers for generation of the homologous construct (5’-3’) included:

LA FW: GGCCATAGCGGCCTCCATCCTTTGGGGGAAAATTGC

TAG RV: TCCGCCGCCAGACCCTCCGCCCTTTTCGAACTGTGGGTGGCTCCATGCGGATTTGTCGTCATCATCCTTGTAGTCCATGGTGGCACCTGCTTCCAACCCGC

TAG FW: TTCGAAAAGGGCGGAGGGTCTGGCGGCGGATCTGGAGGGAGTGCCTGGAGCCATCCCCAGTTTGAGAAAGGCAGCGCTAGTGGCAGCTGTCAGGGGTGC

LA RV: GGCGCGCCAAAGGATATGGGAGTTTGCGGCTCC

RA FW: GGCCGGCCCCTTAGGGCAGAAAGCAGCTGAG

RA RV: CTCGAGTCATAAGACATCACTTTCTTTAG.

1x10^6^ hiPSCs were nucleofected with 3μg of each TALEN pair and 8μg homologous construct using an Amaxa I nucleofector and the corresponding stem cell transfection kit (Lonza). Single cells were plated on MEF coated plates cultured in hES medium for one week and selected with 250 μg/ml Neomycin (Biochrom) for additional two days. Resistant clones were transferred mechanically on MEF coated 12 well plated for further characterization. DNA was isolated using the DNA Blood and tissue isolation kit (Qiagen) and used for sequencing and DNA genotyping. The SF-TAP TAG Sequence was the following: 5’ATGGACTACAAGGATGATGACGACAAATCCGCATGGAGCCACCCACAGTTCGAAAAGGGCGGAGGGTCTGGCGGCGGATCTGGAGGGAGTGCCTGGAGCCATCCCCAGTTTGAGAAAGGCAGC 3’. Primers used to assess successful targeting are reported here: LRRK2 FW: CCCTGCCGGTTCCCTGAG; LRRK2 REV: CCCCTCCTTACATTTGCAAA.

### Quantitative RT-PCR (qRT-PCR)

Total cellular RNA was extracted using the RNeasy Mini Kit (Qiagen) according to manufacturer instructions. RNA isolated from cultured cells was reverse-transcribed using M-MLV Reverse Transcriptase (USB Corporation) with oligo-dT_16_ primers (Metabion) for 1 h at 42°C, followed by 10 min at 65°C. qRT-PCR was performed with a LightCycler LC 480 (Roche) using SYBR green PCR master mix (Life Technologies). Cycling conditions included denaturation for 10 min at 95°C, followed by 40 cycles alternating 15 sec at 95°C and 60 sec at 60°C. Fold changes in gene expression were calculated using the 2^−2Δ^ method, normalized to biological reference samples and using *GAPDH* or *HMBS* as housekeeping gene. See S1 Table.

### Neurite outgrowth assay

For neurite outgrowth, smNPCs were differentiated to mDANs as described above. After 2 days in maturation medium, 3–4 small neuronal clusters were scraped off and transferred onto a 96-well plate (Greiner) previously coated with PLO-laminin (Sigma). Neuronal clusters were incubated overnight at 37°C in the presence of 5% CO_2_. Neurite outgrowth was measured the next morning using an Axiovert 200M microscope endowed with a live-cell heating chamber. Pictures were taken at 20x magnification every 9 minutes over a total time of 3 hours, and analyzed with ImageJ using the plug-in MTrackJ. To assess the effects of the LRRK2 inhibitor CZC-25146 on outgrowing neurites, neurons were pre-treated with the compound before picking, and subsequently transferred to CZC-25146 containing medium in the final plate. Overall incubation time with CZC-25146 at a concentration of 2 μM was 24h. Sequential images were acquired and processed as described above, and final values indicating the length covered by the outgrowing neurite over time, corresponded indirectly to neurite outgrowth velocity.

### Western blot analysis

Whole cell lysates were usually generated in RIPA Buffer (Santa Cruz & Biomol) supplemented with the provided protease inhibitors, as well as the phosphatase inhibitor PhosStop EASyPack (Roche). For LRRK2 experiments, proteins were extracted using Tris-buffered Saline (TBS) with 0.5% NP40 protein extraction buffer containing protease and phosphatase inhibitors (Roche) at 4°C. Either 15 or 30 μg of each protein lysate were mixed with 5x Laemmli buffer and loaded on 4–15% SDS PAGE separation gels after incubation at 95°C for 5 min. Blotting was performed on nitrocellulose (semi-dry blot) or PVDF (wet blot for LC3B and all proteins larger than 90 kDa) membranes. Total protein stain (Ponceau) was performed to assess proper transfer. Membranes were blocked for 1 h in 5% milk in TBS-T 1x, and subsequently incubated with the primary antibody. The following primary antibodies were used: LC3B (Novus Biologicals, NB100-2220), α-synuclein (BD Bioscience, 610787), ATP5A (Mitosciences, AbCam, 14748), TOM20 (Sant Cruz Biotechnology, sc-11415), β-Actin (Sigma Aldrich), GAPDH (Calbiochem, CB1001), Vinculin (Vinculin, V9131), LAMP1 (DSHB, H4A3), ERK1/2 and P-ERK1/2 (Cell Signaling Technolgy, 4370, 4696), actin (SIGMA, A5441), LRRK2 (MJFF, c41) were used. Membranes used for aSYN western blot were first fixed in 4% (w/v) paraformaldehyde and washed before blocking. After 3x washing steps with TBS-T, membranes were incubated with HRP-coupled secondary antibodies purchased from GE Healthcare. Densitometry from Western blot was performed using the Image J software (Wayne Rasband; National Institutes of Health, USA).

### Immunocytochemistry staining

For immunofluorescence, cells were fixed for 20 minutes with 4% paraformaldehyde (EM Sciences) in PBS. Permeabilization and blocking were performed in a single step using PBS (Lonza) supplemented with 0.1% Triton X-100 (Sigma), 10% FCS (GE Healthcare) and 1% BSA. Primary antibodies were applied overnight at 4°C in PBS containing 0.1% BSA. Subsequently, cells were incubated with secondary antibodies for 1 h at room temperature and ultimately washed 3x, including a Hoechst (Thermo Fisher Scientific) counterstaining for nuclei in the second washing step. Primary antibodies used in this study included: goat anti-Sox2 sc-17320 1:200 and mouse anti-Oct4 sc-5279 1:200 (both Santa Cruz), mouse anti-SSEA4 MC-813-70 1:200 (DSHB), goat anti-Sox1 AF3369 1:400, mouse anti-Nestin MAB1259 1:500, (both R&D Systems), rabbit anti-TH sc-14007 1:300 (Santa Cruz), mouse anti-Tuj1 MMS-435P 1:1000 (BioLegend). For fluorescence microscopy analysis, secondary antibodies conjugated to Alexa-488, 568 or 647 (1:1000, Thermo Fisher Scientific) were used. Cells were imaged on an inverted Apotome Zeiss Axio/Observer Z1 fluorescence microscope.

### Statistical analysis

Measurements were usually obtained from at least 3 independent experiments. Statistical significance was calculated using the Mann-Whitney test. Variance was assessed by Levene’s test. Corresponding results are reported under the column “variance” in S2 Table.

## Results

### Generation and differentiation of iPSCs

**Fig 1:**
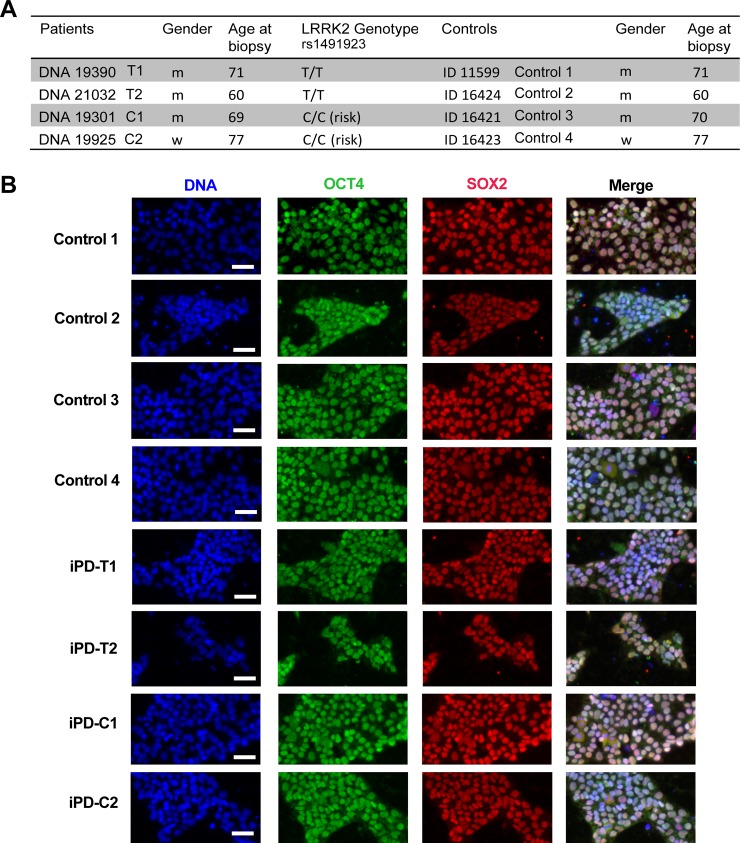
Overview of the lines included in this study. (A) iPSCs were successfully generated from 4 healthy individuals as well as 4 iPD patients (2 carrying the T/T LRRK2 genotype and 2 with the C/C LRRK2 SNP RS1491923). (B) The iPSCs were characterized for pluripotent stem cell marker expression by immunofluorescence. Scale bar is 50 μm. See also S1 and S2 Figs.

iPSCs were derived from four patients with iPD as well as four age and gender matched controls (Fig 1A). All lines had wild type GBA and were analyzed for the most important common risk factors for PD, including SNCA (G/G, rs356219) and MAPT (A/A, rs393152) [10, 23, 24]. Two iPSC lines contained the C/C risk allele genotype at rs1491923 and were designated iPD-C1 and iPD-C2. Two iPSC lines had the T/T non-risk allele genotype and were labeled as iPD-T1 and iPD-T2. The four control iPSC lines were designated Control1, Control2, Control3, and Control4. Dermal fibroblasts from skin punch biopsies of patients with PD or healthy controls were expanded and either infected with Sendai virus or transfected with episomal expression vectors for OCT4, SOX2, C-MYC, and KLF4 (Panel A in S1 Fig). Individual clonal iPSC lines were expanded and confirmed to be transgene and integration free by quantitative RT-PCR (Panels B and C in S1 Fig). This assay demonstrated that all iPSC lines expressed endogenous OCT4, SOX2, KLF4, and C-MYC at levels comparable to previously derived and characterized iPSCs [12] (Panels D and E in S1 Fig), and immunostaining confirmed expression of the pluripotency marker proteins OCT4 and SOX2 (Fig 1B). Metaphase spreads or SNP microarray analysis confirmed euploidy of all lines (Panel A in S2 Fig).

**Fig 2:**
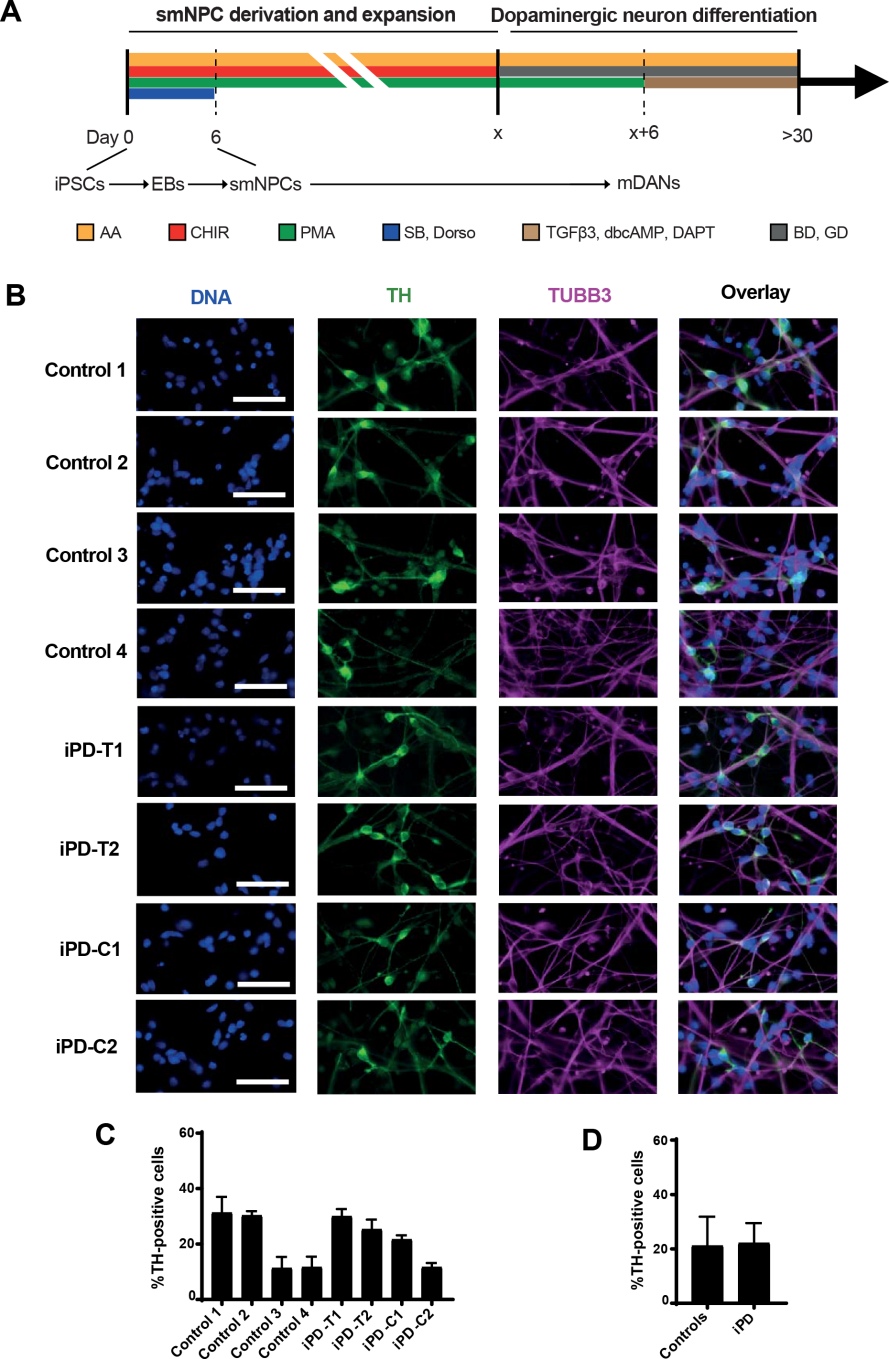
Produced iPSCs can be differentiated to mDANs. (A) Schematic representation of the differentiation protocol used in this study. AA indicates ascorbic acid; CHIR indicates CHIR99021, a GSK3 inhibitor; SAG indicates Smoothened agonist; SB indicates SB43152, an inhibitor of TGFβ receptors; Dorso indicates dorsomorphin, an inhibitor of BMP receptors; BD indicates BDNF; GD indicates GDNF. (B) Fluorescent micrographs of iPSC-derived mDANs for the indicated markers. Scale bar is 50 μm. (C) Evaluation of mDAN average differentiation efficiency for healthy controls and iPD lines. Efficiencies for each single line are shown in panel (D). TH positivity identifies *bona fide* mDANs. See also S3 Fig.

Characterizing neurons differentiated from 8 different iPSC lines in parallel requires a protocol that is robust, well-established, easy to handle, and reproducible between cell lines. For these reasons, iPSCs were initially differentiated into small molecule-induced neural progenitor cells (smNPCs), which undergo limitless self-renewal in the presence of the small molecules purmorphamine (PMA) and CHIR99021 [25] (Fig 2A). Immunostaining confirmed expression of the neural markers SOX1, NESTIN, and PAX6 (S3 Fig). As described previously, smNPCs exposed to the neurotrophins BDNF and GDNF for 6 days (Fig 2A), followed by maturation in BDNF, GDNF, TGFβ3, cAMP, and DAPT-containing medium resulted in neuronal cultures where an average of about 20% of cells in both controls and iPD lines expressed Tyrosine Hydroxylase, a marker of dopaminergic neurons (DANs; Fig 2B–2D). Previously, we demonstrated that neurons differentiated using this protocol are electrophysiologically functional [25]. SNP genotyping demonstrated that the genotype of neurons differentiated from each iPSC line correctly matched their original patient fibroblasts (Panel B in S2 Fig).

### Validation of LRRK2 inhibition using an additional gene-edited knock-in iPSC line

Because mutations in the LRRK2 gene are the most common cause of fPD, and different SNPs in the LRRK2 locus have been associated with an increased risk for developing iPD, one question we attempted to address was whether LRRK2 inhibition could be beneficial in iPSC-derived DANs. First, we measured basal LRRK2 levels using our system. According to available databases collecting information on gene expression from different tissues, both RNA and protein levels of LRRK2 across the brain are considerably lower compared to other tissues, such as heart, liver, kidney and lungs [26]. We performed qRT-PCR for *LRRK2* mRNA on cultures of DANs differentiated from Control and iPD-iPSCs. LRRK2 was expressed in all lines at levels that were barely detectable with our assay, and no significant differences were observed between Controls and iPD lines (Panel A in S4 Fig). Western blot analysis also showed very low levels of expression, which proved cumbersome to quantify (Panel B in S4 Fig), consistent with previous reports indicating that DANs contain relatively low LRRK2 protein compared to other cell types [26, 27]. There was no obvious difference between healthy controls and iPD lines (Panels A and B in S4 Fig).

**Fig 3:**
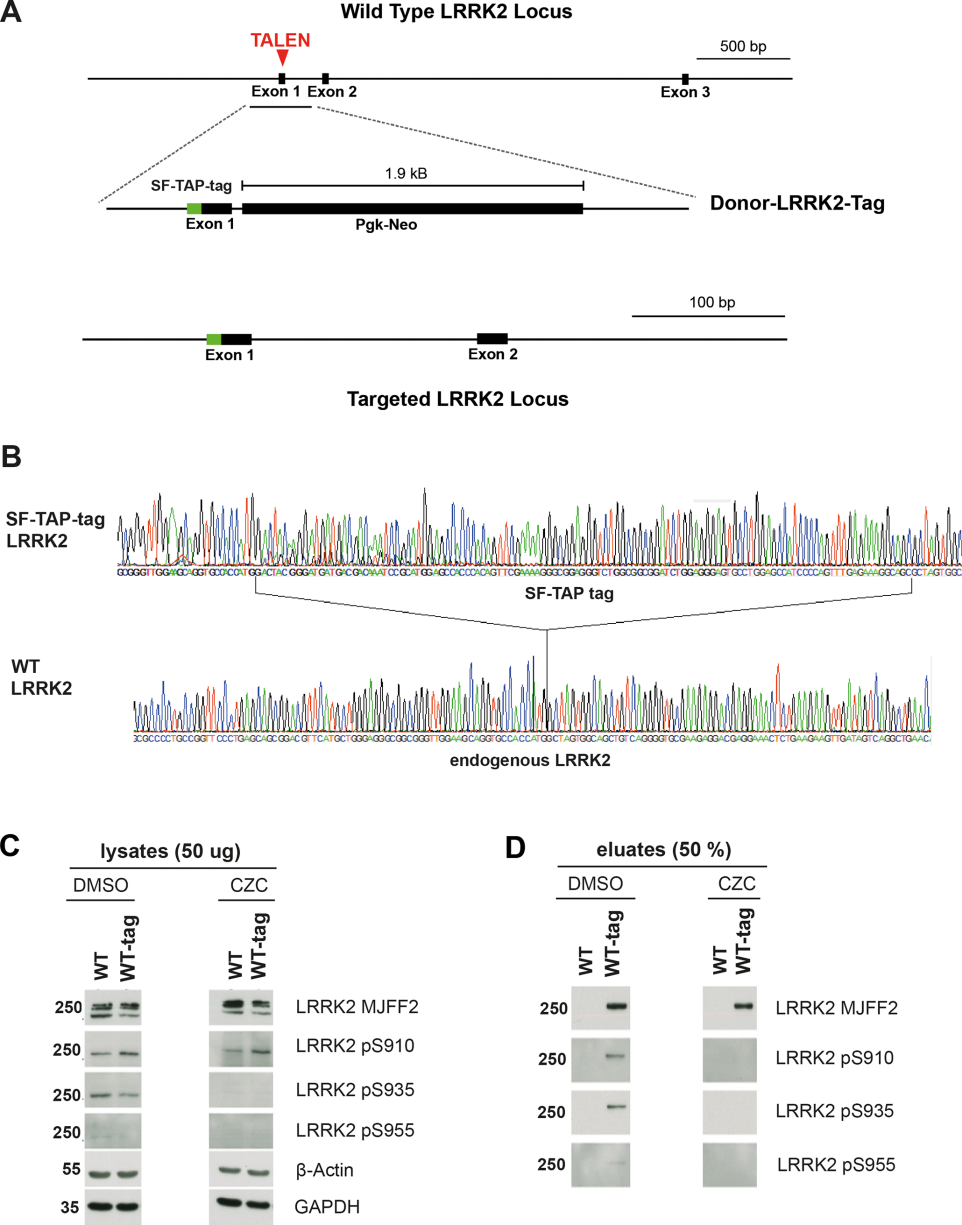
Analysis of LRRK2 levels. (A) Strategy to insert an SF-TAP tag at the N-terminus of exon 1 of LRRK2. A TALEN pair targeting exon 1 of LRRK2 was administered along with a donor construct containing homology arms flanking the SF-TAP tag (green box) and a neomycin resistance cassette. Red arrows indicate the genomic TALEN cutting site; black boxes represent exons or the neomycin resistance cassette, respectively. (B) Sanger sequencing showing integration of the SF-TAP tag into the wild type LRRK2 locus. (C and D) Detection of endogenous LRRK2 and inhibition using CZC-25146 is better validated by selectively immunoprecipitating SP-TAP tagged LRRK2. Western blot analysis for the indicated antigen was conducted on iPSC-derived mDANs on both whole cell lysates (C) and IP products (D). See also S4 Fig.

Phosphorylation of serines 910, 935, 955, 973, and 1292 of the LRRK2 protein is sensitive to LRRK2 inhibition, and loss of phosphorylation at these sites is routinely used to monitor target engagement of small molecule LRRK2 inhibitors [28, 29]. Because of the low LRRK2 expression levels, we adopted an alternative method to assess target engagement, and performed affinity purification of an additional set of lines carrying a LRRK2-tag, followed by detection of ser910 and Ser935 phosphorylation. Previously, we successfully used the Strep/Flag tandem-affinity purification (SF-TAP) tag to purify transgenic LRRK2 [30]. This method consists of tagging a target protein with a streptavidin-tag as well as a FLAG moiety optimized for rapid as well as efficient tandem affinity purification. TAL effector nucleases (TALENs) were designed against a target upstream of the translational start site to knock-in and fuse the SF-TAP tag to the N-terminus of LRRK2 in WT iPSCs (Fig 3A). Sequencing confirmed the correct integration of the SF-TAP tag (Fig 3B). Western blotting and qRT-PCR confirmed expression of the tagged LRRK2 protein (Panels D and F in S4 Fig). Quantitative RT-PCR and western blotting confirmed that the SF-TAP tag did not effect LRRK2 expression (Panels E and G in S4 Fig).

CZC-25146 (hereafter abbreviated CZC) is a potent and selective LRRK2 inhibitor [31] known to inhibit LRRK2 activity in iPSC-derived neurons [32]. To test LRRK2 inhibition by CZC, we cultured iPSC-derived neurons for 24 hours with 3 μM CZC. Lysates were harvested and used for affinity purification followed by western blot analysis for phospho-ser910, phospho-ser935, phosho-ser955, and total LRRK2. As expected, unpurified lysates showed inconsistent results due to low LRRK2 levels as well as unspecific antibody binding (Fig 3C). After affinity purification, phospho-ser910, phospho-ser935, and phospho-ser955 were detected only in samples from untreated control cultures and were not detected in samples from cultures treated with CZC (Fig 3D). These results demonstrate that CZC potently inhibits endogenous LRRK2 kinase activity, and, therefore, CZC was chosen to test the effects of LRRK2 inhibition in all future experiments.

### Reduced neurite outgrowth in LRRK2 G2019S is not corrected by CZC-25146

**Fig 4:**
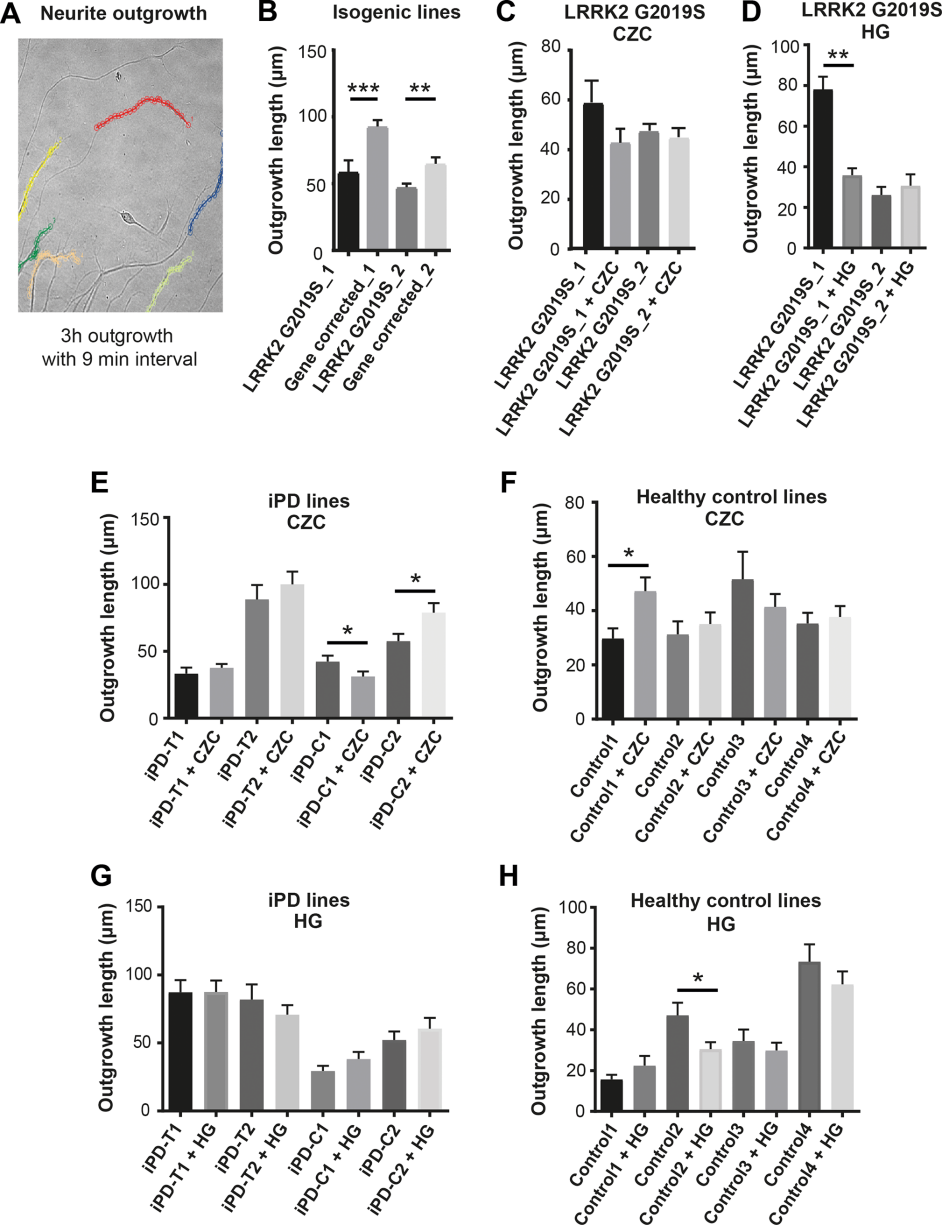
Neurite outgrowth analysis. (A) Example of neurite outgrowth experimental setup. 5 positions per line were selected and imaged every 9 minutes over a period of 3 hours. Neurite length was assessed using Fiji’s plugin mTrackJ. (B) Neurite outgrowth in isogenic LRRK2 G2019S and gene-corrected WT iPSC-derived DANs. (C) Neurite outgrowth in neurons with *LRRK2* G2019S in the presence or absence of the LRRK2 inhibitor CZC-25146. (D) Neurite outgrowth in LRRK2 G2019S lines with and without the LRRK2 inhibitor HG-10-102-01. (E) Neurite outgrowth in iPD lines with and without the LRRK2 inhibitor CZC-25146. (F) Neurite outgrowth in healthy control lines with and without the LRRK2 inhibitor CZC-25146. (G) Neurite outgrowth in iPD lines with and without the LRRK2 inhibitor HG-10-102-01. (H) Neurite outgrowth in healthy control lines with and without the LRRK2 inhibitor HG-10-102-01. Graphs show mean with standard error of mean (S.E.M). Significance was calculated using Mann Whitney test. * indicates p<0.05, ** indicates p<0.01, *** indicates p<0.001. n = 20 neurites analyzed from neurons of 3 independent cultures.

Previously, we demonstrated that neurons with LRRK2 G2019S show significantly reduced velocity of neurite outgrowth compared to isogenic WT controls [12]. This phenotype was, therefore, used to evaluate the effects of LRRK2 inhibition in our cohorts of iPSC-derived neurons (Fig 4A). As previously published [12], neurons with G2019S showed significantly less neurite outgrowth compared to isogenic gene-corrected controls (Fig 4B). This difference only existed when each line was compared to their respective isogenic control, but not when genetically different WT and/or LRRK2-mutant cell lines were pooled into two separate groups and then compared. To test the effects of LRRK2 inhibition on neurite outgrowth, iPSCs with *LRRK2* G2019S were differentiated into neurons and neurite outgrowth was measured with and without CZC. We only compared outgrowth using one cell line treated compared to the same cell line untreated only. Unexpectedly, no significant improvement was detected upon treatment, indicating that LRRK2 kinase activity inhibition does not affect neurite outgrowth (Fig 4C). Similarly, when we tested LRRK2 kinase inhibition by CZC on all the cell lines involved in this study, we did not observe any consistent trend either in neurons differentiated from iPD patients, even when grouped by the LRRK2-RS1491923 genotype, or in age and gender matched controls (Fig 4D and 4E). These data suggest that LRRK2 kinase activity does not play a significant role in regulating the velocity of neurite outgrowth. These results were also confirmed using an additional LRRK2 inhibitor, HG-10-102-01 (HG), which, similar to CZC, induced variable responses in all lines tested without a consistent pattern of either amelioration or deterioration of neurite outgrowth. Opposite effects of CZC or HG on the various cell lines could be due to off-target kinases being inhibited as well as different polymorphisms in the genetic background.

### LRRK2-RS1491923 may be associated with variation in autophagy markers

**Fig 5:**
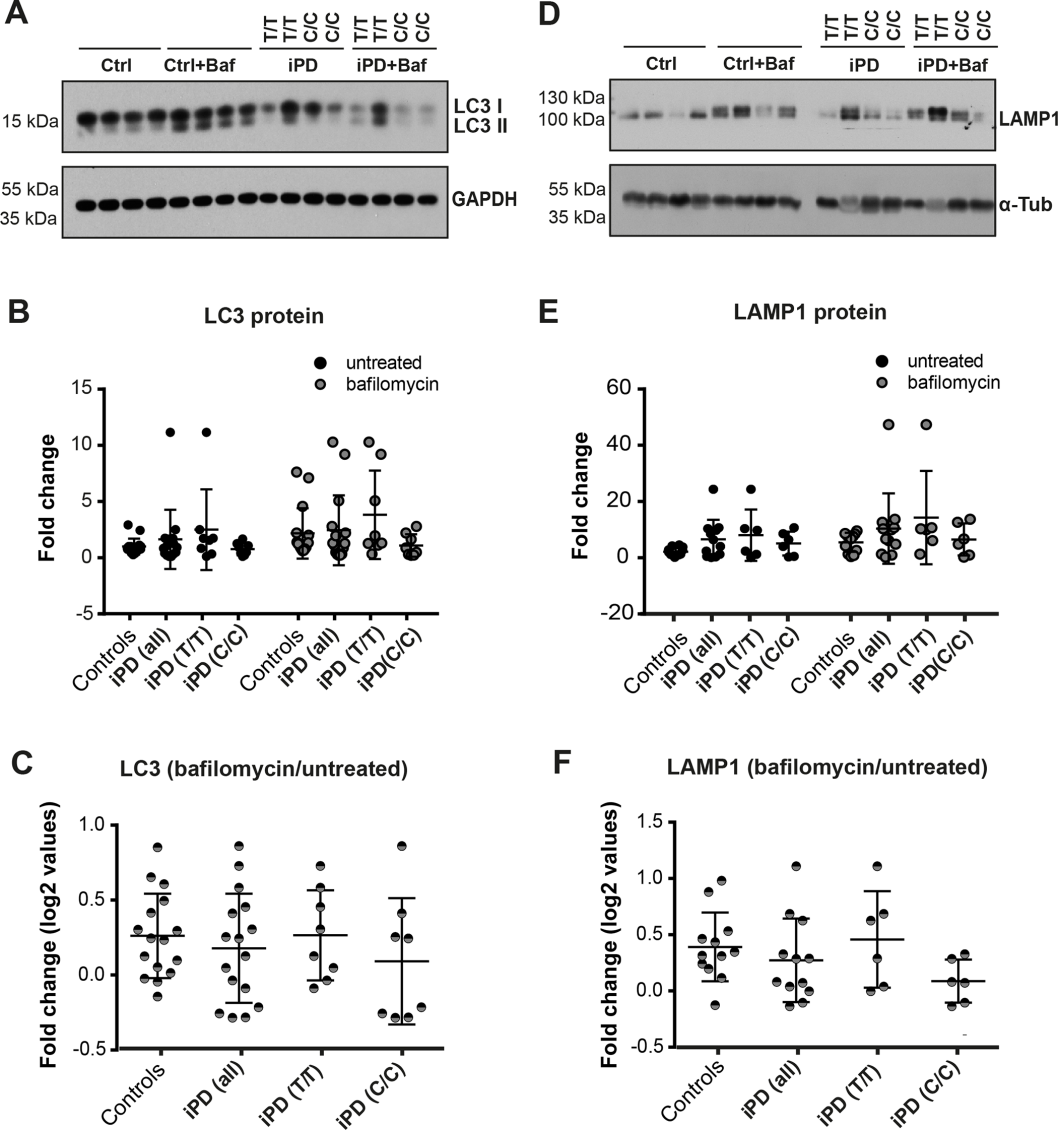
Investigation of autophagy. (A) Representative western blot of LC3B-I, LC3B-II and GAPDH loading control in DANs derived from 4 iPD patients and 4 healthy controls. Labels C/C (risk allele) and T/T (non-risk allele) denote the genotype of iPD patients at LRRK2-RS1491923. mDANs were treated with a DMSO vehicle control or 100 nM bafilomycin (baf) for 24 h. (B) Western blots from three independent experiments (n = 3) were quantified by densitometry and LC3B-II normalized to a loading control. All measurements are shown. Data is grouped as healthy vs iPD as well as iPD stratified for T/T and C/C genotype. (C) Ratio of LC3B-II (normalized to loading control) following bafilomycin treatment/untreated. (D) Representative western blot of LAMP1 and α-tubulin loading control in mDANs derived from iPD patients and healthy controls. (E) Western blots from three independent experiments (n = 3) were quantified by densitometry and LAMP1 normalized to a loading control. All data is shown, including data stratified for T/T and C/C genotype. (F) Ratio of LAMP1 (normalized to loading control) following bafilomycin treatment/untreated.

Because defects in autophagy have been linked to LRRK2 and described to contribute to PD pathogenesis [13], we assessed autophagy using western blot analysis for LC3B-II normalized to GAPDH in cultures of iPSC-derived neurons. LC3B is a soluble protein that during autophagy is lipidated (LC3B-II) and specifically recruited to autophagosomal membranes, thus acting as a marker for the autophagosomal compartment. The only difference we observed was that the variance in basal LC3B-II levels in iPSC-derived neurons from iPD patients was greater than in neurons from control iPSCs (Fig 5A and 5B, S2 Table). There was a trend towards higher LC3B-II basal levels in iPD cultures compared to controls, but this was not statistically significant (Fig 5B). Next we assessed the levels of LC3B-II (normalized to a loading control) before and after stimulation of LC3B-II accumulation induced by bafilomycin, which inhibits the fusion between autophagosomes and lysosomes. Bafilomycin treatment caused increased LC3B-II accumulation as expected (Fig 5C). Overall, there were no significant differences between autophagic flux between iPD and the control group (Fig 5C). However, when we grouped the iPD cell lines based on LRRK2 risk, we found that autophagic flux was slightly reduced in C/C LRRK2-RS1491923 neurons compared to T/T LRRK2 rs1491923 and to healthy controls (Fig 5C).

Next, we assessed the induction of increased lysosome levels using western blot analysis for LAMP1. Again, iPD-iPSC-derived neurons showed higher variance compared to healthy controls (Fig 5D and 5E, S2 Table). We also observed a trend toward higher average LAMP1 levels in neurons from iPD-iPSCs compared to controls, although this was not statistically significant (Fig 5E). Additionally, neurons from iPD and healthy iPSCs were treated with 100 nM bafilomycin for 24 hours, and, as expected, bafilomycin treatment increased the steady state levels of LAMP1 in all groups compared to untreated (Fig 5E). We found that the ratio of LAMP1 in bafilomycin treated versus untreated neurons was decreased in the iPD cohort versus healthy controls (Fig 5F). This effect was recapitulated when we grouped the lines for the C/C LRRK2-RS1491923 risk. These data suggest that the C/C LRRK2-RS1491923 risk in iPD could be stratified for a possible autophagy endophenotype.

### LRRK2-RS1491923 may be linked to subtle alterations in mitochondrial turnover

**Fig 6:**
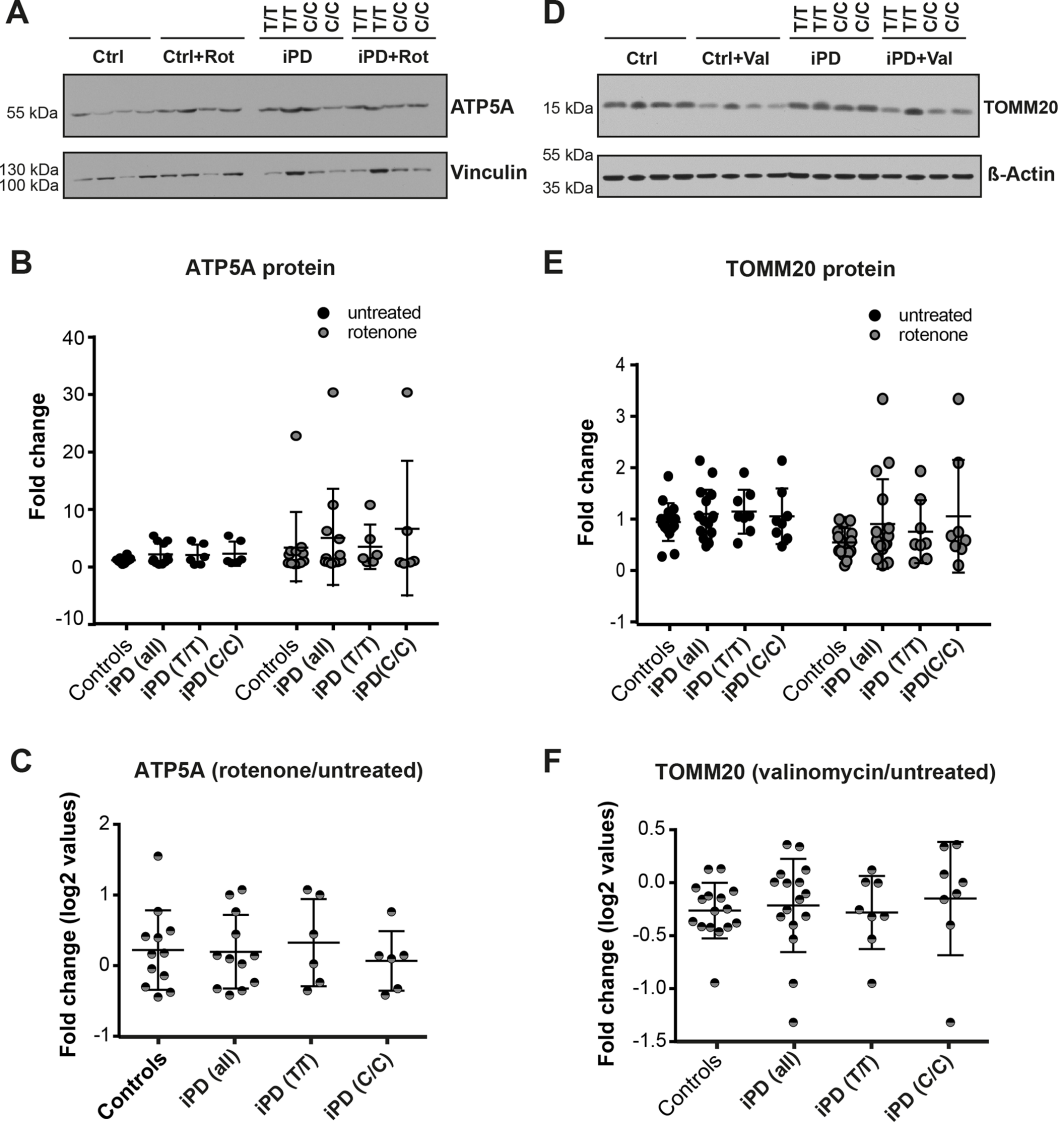
Quantification of mitochondrial protein levels. (A) Representative western blot of ATP5A and Vinculin loading control in mDANs derived from 4 iPD patients and 4 healthy controls. Labels C/C (risk allele) and T/T (non-risk allele) denotes the genotype of iPD patients at LRRK2-RS1491923. DANs were treated with a DMSO vehicle control or 100 nM rotenone (rot) for 24 h. (B) Western blots from three independent experiments (n = 3) were quantified by densitometry and ATP5A normalized to a loading control. All data points are reported. Data is grouped as healthy vs iPD as well as iPD data stratified for T/T and C/C genotype. (C) Ratio of ATP5A (normalized to loading control) following rotenone treatment/untreated. (D) Representative western blot of TOMM20 and β-actin loading control in DANs derived from iPD patients and healthy controls. mDANs were treated with a DMSO vehicle control or 1μM valinomycin (val) for 24 h. (E) Western blots from three independent experiments (n = 3) were quantified by densitometry and TOMM20 normalized to a loading control. Data stratified for T/T and C/C genotype for both the untreated and rotenone treated condition are also shown. (F) Ratio of TOMM20 (normalized to loading control) following valinomycin treatment/untreated.

Mitochondria play a critical role in PD pathogenesis. Dysfunction of mitochondria linked to bioenergetic defects, damage to mitochondrial DNA, changes in the dynamics of mitochondrial fusion, fission, size and morphology, as well as alterations in mitochondrial trafficking are implicated in PD [33, 34]. To investigate mitochondrial mass, we initially characterized the levels of the mitochondrial markers TOMM20 (mitochondrial import receptor subunit) and ATP5A (a subunit of complex V of the respiratory chain) in iPSC-derived neurons using western blot analysis. We found that both markers were expressed at very similar levels in all cell lines under normal conditions (Fig 6A–6D). When we induced mitochondrial stress using the complex I inhibitor rotenone, the steady state levels of ATP5A were slightly increased in all lines (Fig 6B and 6E), consistent with the idea that respiratory complex subunits are upregulated as a mechanism to compensate for mitochondrial damage. However, we detected no clear differences between iPD and control groups in regard to the ratio of ATP5A levels rotenone treatment versus untreated (Fig 6E).

*PINK1* and *PRKN* (encoding PARK2 also known as PARKIN) mutations linked to PD have been shown to disrupt mitochondrial autophagy (mitophagy) in response to ionophores [34–36]. Because LRRK2 has also been described to impact on mitochondrial dynamics and function [34], we tested mitochondrial autophagy in iPSC-derived neurons. To do this, differentiated neurons were treated with 1 μM valinomycin for 24 hours [37] followed by western blot analysis for TOMM20 and the housekeeping gene β-actin (Fig 6D and 6F). Valinomycin is a dodecadepsipeptide that forms a lipid-soluble complex with K+ that leads to the depolarization of mitochondria, which induces mitophagy to clear out non-functional mitochondria. The ratio of valinomycin treated to untreated TOMM20 levels normalized to β-actin was used to minimize variation from the genetic background (Fig 6F). Almost all control neurons had a ratio of valinomycin treated/untreated of less than 1, and the overall average was approximately 0.57, indicating that valinomycin reduced levels of TOMM20 compared to untreated cells. This is consistent with the clearance of defective mitochondria following mitochondrial depolarization. However, while the degradation of matrix proteins and mitochondrial DNA occurs almost exclusively by mitophagy, outer mitochondrial membrane proteins, such as TOMM20, can also be rapidly degraded by the proteasome under mitophagy-inducing conditions [38, 39]. Therefore, we cannot exclude that the reduction in TOMM20 protein levels upon valinomycin treatment may also be associated with increased degradation of TOMM20 by the proteasome. In contrast to control neurons, the average ratio of iPD cultures was about 1.1, suggesting a defect in clearing mitochondria following depolarization, or in mitochondrial outer membrane protein degradation via the proteasome (Fig 6F). Consistent with our pervious results with LC3B-II and LAMP1, we observed greater variance in iPD-derived neurons compared to controls (Fig 6D and 6F). When iPD cultures were grouped by their LRRK2-RS1491923 genotype, we observed that the T/T genotype showed an average ratio of about 0.6, indicating that mitochondrial autophagy/proteasome-mediated degradation was still taking place similarly to healthy cells (Fig 6F). In contrast, neurons carrying the C/C genotype showed an average ratio of about 1.6 (Fig 6F), suggesting a potential defect in mitochondrial autophagy/proteasome-mediated degradation associated with the C/C LRRK2-RS1491923 risk allele.

We previously reported increased ERK signaling in G2019S LRRK2 neurons compared to an isogenic control [12], and at least one report demonstrated that LRRK2 G2019S dysregulates autophagy through ERK activation [40]. For these reasons, we assessed the levels of phosphorylated ERK1/2 (pERK) in control and iPD neurons. Here, we found no differences in pERK normalized to total ERK between the control and iPD groups, although the variation across the groups was high (Panels A and B in S5 Fig). The LRRK2 inhibitor CZC had the overall effect of slightly increasing pERK levels across all groups (Panel C in S5 Fig), irrespective of phenotype or genotype. This suggests that the C/C LRRK2-RS1491923 risk allele may induce autophagy alterations independently of increasing pERK, which is distinct from G2019S.

### LRRK2 inhibition may influence aSYN protein levels in neurons from iPD patient iPSCs

**Fig 7:**
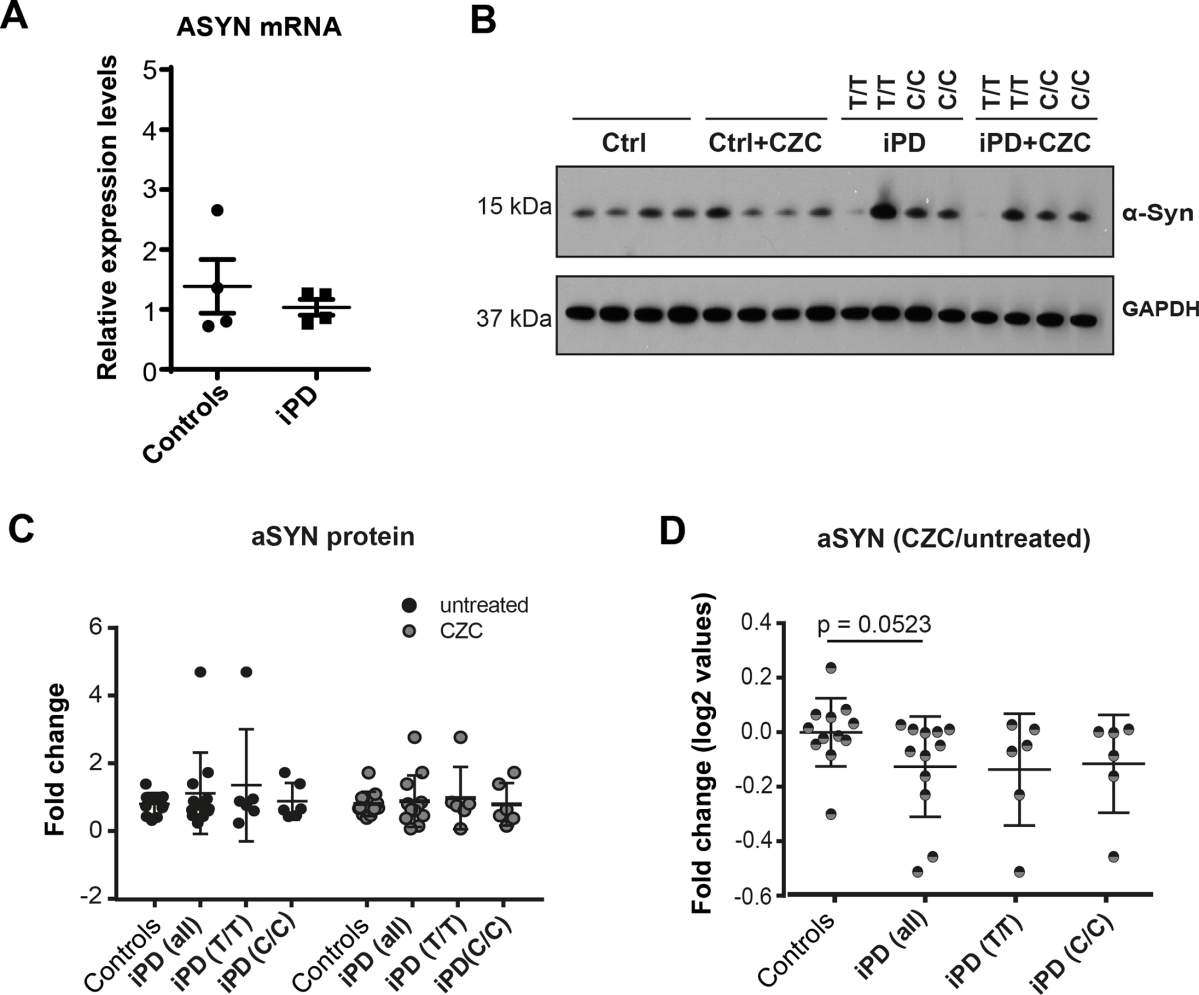
Analysis of aSYN levels. (A) qRT-PCR data showing aSYN mRNA expression levels in healthy controls and iPD lines. No significant differences are detected between the groups. (B) Representative western blot of aSYN and GAPDH loading control in DANs derived from 4 iPD patients and 4 healthy controls. Labels C/C (risk allele) and T/T (non-risk allele) denote the genotype of iPD patients at LRRK2-RS1491923. mDANs were treated with a DMSO vehicle control or 2 μM CZC (CZC) for 24h. (C) Western blots from three independent experiments (n = 3) were quantified by densitometry and aSYN normalized to a loading control. Graphs show all individual measurements. Stratification for T/T and C/C genotype is also included. (D) Ratio of aSYN (normalized to loading control) following CZC treatment/untreated. Significance was calculated using Mann-Whitney test.

aSYN pathology is a hallmark of PD, has been linked to defects in mitochondrial autophagy [41], and increased aSYN levels have been observed in DANs from *G2019S* LRRK2 Parkinson’s disease patients compared to isogenic controls [12]. For these reasons, we investigated aSYN levels in our iPD and control lines. First, we measured *aSYN* RNA levels in cultures of DANs differentiated from control and iPD lines using quantitative RT-PCR (Fig 7A). However, we observed no statistically significant difference.

Next, we evaluated the levels of aSYN protein in iPD-derived neurons versus healthy controls by western blot, and, similar to our qRT-PCR results, we observed no statistically significant difference (Fig 7B and 7C). This was not surprising, because iPD pathogenesis remains to be elucidated and it is not expected to observe striking phenotypes. In addition, the high degree of biological variability between the cell lines may contribute to masking subtle phenotypes.

The G2019S mutation increases LRRK2 kinase activity [42–44] as well as aSYN levels [12], and increased aSYN levels directly induce PD pathogenesis [45, 46]. Even though we were not able to detect a significant increase in aSYN in our iPD lines, our final experiment was to test the effects of LRRK2 inhibition on aSYN protein levels, knowing that a reduction in LRRK2 kinasic activity should result in an amelioration of aSYN levels. Cultures of differentiated neurons were treated with and without 2 μM CZC for 24 hours and aSYN levels were assessed using western blot analysis and normalized to GAPDH. In spite of some variability, the average ratio of aSYN levels after CZC treatment versus untreated in cultures of control neurons was approximately 1, indicating that aSYN levels were unchanged by CZC (Fig 7D). In contrast, neurons from iPD samples treated with CZC showed a reduction of aSYN, which almost reached statistical significance (p = 0.0523) (Fig 7D). Interestingly, aSYN levels appeared as reduced in iPD-derived neurons with both LRRK2-RS1491923 C/C and T/T. These results suggest that CZC treatment may have unmasked a very subtle but potentially interesting phenotype. Validation of this result would indicate that reducing relative aSYN levels through LRRK2 inhibition might be beneficial to all iPD patients, as it would reduce their risk of aSYN pathology. However, further experiments incorporating larger sample numbers are required to establish a firmer link between LRRK2 inhibition and aSYN protein level reduction.

## Discussion

Our current knowledge of the molecular mechanisms of PD pathogenesis largely derives from familial forms, which are caused by recessive mutations, such as in *PARKIN* and *PINK1*, as well as dominant mutations, including in *SNCA* and *LRRK2* [47]. However, approximately 90% of PD cases are idiopathic. It has been suggested that the total heritability of PD is about 30% [3], and GWA studies have identified common polymorphisms at multiple loci that are associated with increased risk of PD pathogenesis [24]. Models of iPD are urgently needed in order to understand how much overlap these familial genetic mechanisms have with sporadic cases as well as to test possible therapeutics that would benefit the majority of PD patients. However, the lack of a single causal mutation has made it extremely difficult to generate models of iPD.

The characteristics of iPSCs make them promising tools for generating patient-specific disease models, possibly also from iPD patients lacking a specific or known causal mutation. This paves the way for *in vitro* investigation of complex and multifactorial neurodegenerative diseases, such as PD. Importantly, since iPSCs are expandable, they are also ideal for generating scalable iPD models for drug testing.

In this work, we explored the possibility to perform iPD *in vitro* disease modeling using iPSCs. This study is extremely important, as it represents one of the first attempts to apply iPSC technology to the most prevalent, yet least investigated, form of PD. One major challenge of iPSC-based models is finding a sufficiently strong pathological phenotype for disease modulation. A complicating factor is that iPD represents a collection of different disease subtypes, where multiple mechanisms of pathogenesis are likely to be involved [48]. Consistent with this idea, our results showed that the simple grouping of data from iPD patient neurons results in very large variations for the investigated readouts. However, genotype-based stratification may be possible, and would provide a smarter way to interpret the results. Our data suggests that iPD samples with LRRK2-RS1491923 C/C may have defects in autophagy and mitochondrial protein clearance, which corroborates at least one recent report [16]. However, achieving reproducible statistical significance with genotype stratification will require a large number of iPSC lines. For example, GWA studies typically include thousands of patients. In contrast, iPSC-based disease modeling experiments only use a handful of lines because they are performed manually and can be very cumbersome. Ongoing efforts to automate disease modeling will be critical to increasing the sample sizes for modeling idiopathic diseases, such as iPD [49], in order to obtain results with sufficient robustness to enable drug discovery. Increasing the number of analyzed iPSC-derived neuronal lines would also help compensate for the intrinsic variability between cultures of iPSC-derived neurons, which critically contributes to noisy data.

LRRK2 inhibitors are being developed as possible PD therapeutics, and multiple experiments show that they ameliorate pathology caused by G2019S, including in iPSC-derived neurons [12, 19, 50]. Our iPD model offered a platform to test whether LRRK2 inhibition could be an effective method for ameliorating pathological phenotypes in iPD neurons. Our data suggested that LRRK2 inhibition may reduce aSYN levels even in iPSC-derived neurons from iPD patients, indicating that these inhibitors could potentially be effective for a wide number of PD patients. These results are preliminary, as the large variability in the datasets linked to the different genetic backgrounds and culture conditions prevented data from reaching statistical significance. In order to reduce the noise, further work will require incorporation of larger sample numbers. Therefore, this approach should be extended in the future, and could prove particularly interesting when incorporated into the design of clinical trials. Indeed, by using iPSC-derived neurons, it would, in principle, be possible to identify patients that are more likely to respond to a specific compound, thereby making clinical testing more effective.

Taken together, we here described a model of iPD based on the use of a small cohort of patient-derived iPSCs as an attempt to investigate Parkinson’s disease in its idiopathic form, for which no significant model has yet been reported.

Although our results did not reach statistical significance due to the reduced sample size, and the detected phenotypes were very subtle as expected in the context of an idiopathic pathology, we posit that our study represents a useful contribution to the PD community, since it highlights the potentials and approachable limitations of our disease modeling tools. Using iPSCs to model iPD undoubtedly represents an appealing strategy, although improvements in data collection and investigation are needed. For instance, consistent with GWAS data, we found large variability in the results, suggesting that iPSC-based studies might need to be similarly structured in order to derive statistically significant data and draw robust phenotypic conclusions. Patterning future iPSC iPD studies on GWA studies may enable the stratification of PD subtypes for drug testing and clinical trials, a critical step toward personalized medicine.

## Supporting information

S1 Fig Reprogramming of adult fibroblasts into iPSCs. (A) Schematic representation of the reprogramming protocol used in this study. Two control and two iPD iPSC lines were generated by Sendai virus infection. The remaining control and iPD iPSC lines were obtained by plasmid transfection. (B) qRT-PCR results showing the absence of viral transgene expression in the generated iPSC lines after passage P7. (C) Agarose gel showing that lines reprogrammed via plasmid transfection do not express plasmidic pluripotency transgenes. A PCR-amplified product is only present in the positive control for the experiment. (D) qRT-PCR results showing successful induction of pluripotency marker expression in target cells after infection. Values are expressed as fold to a control iPSC line. (E) qRT-PCR results showing successful induction of pluripotency marker expression in target cells upon plasmid transfection. Values are expressed as fold to a control human embryonic stem cell line (huES).
(PDF)

S2 Fig Euploidy assessment and paternity test. (A) Metaphase spreads and SNP microarray demonstrating euploid karyotype of the iPSCs generated in this study. Each chromosome with the corresponding B allele frequency and log R ratio is shown. (B) Quality control-paternity test results demonstrating that the genotype of each mDAN line corresponds to that of the putative parental fibroblast cell line. 11599 = Control 1; 16424 = Control 2; 19301 = iPD C1; 16423 = Control 3; 16421 = Control 4; 19390 = iPD T1; 19925 = iPD C2; 21032 = iPD T2.
(PDF)

S3 Fig Analysis of smNPC differentiation. Fluorescent micrographs of iPSC-derived smNPCs for the indicated markers. Scale bar is 50 μm.
(PDF)

S4 Fig Generation of LRRK2 SF-TAP tagged lines and comparison of LRRK2 protein detection in whole cell lysates versus IP products. (A) Generation of the LRRK2 SF-TAP TAG donor construct. Primers containing the SF-TAP TAG sequence were designed to amplify two separate products of the homologous left arm from genomic DNA (PCR 1). An overlapping PCR from those products generated the final homologous left arm containing tagged exon 1 as well as Sfi and AscI restriction sites (orange, blue) (PCR 2). The homolgous right arm was amplified directly amplified from genomic DNA containing the restriction sites Fse and Xho (green, purple) (PCR 3). Left and right arm were then digested and ligated in the pEasyFloxII donor construct. LA = left arm, RA = right arm. (B) DNA agarose gel reveals the heterozygous integration of the SF-TAP tag in the LRRK2 locus. Primers were designed to amplify exon 1 of LRRK2 outside of the targeted region. (C) qRT-PCR analysis proving expression of tagged LRRK2 in DANs. (D) qRT-PCR analysis showing that endogenous LRRK2 expression in LRRK2 tagged and wildtype DANs is not altered. E) Western blot for LRRK2 performed in WT fibroblasts, WT DANs and SF-TAP tagged DANs. Vinculin was used as a loading control. (F) Western blot for LRRK2 performed in WT and SF TAP tagged DANs using actin as a loading control.
(PDF)

S5 Fig Characterization of LRRK2 and ERK levels. (A) qRT-PCR data showing LRRK2 mRNA expression levels in healthy controls and iPD lines. Each point represents average values of duplicates for each individual sample. No significant differences are detected between the groups. (B) Representative western blot of LRRK2 detected in iPD, age and gender-matched controls, and isogenic lines using an anti-LRRK2 antibody. Detection of endogenous LRRK2 in DANs requires protein purification and high concentrations for better resolution. (C) Representative western blot of P-ERK1/2 and total ERK1/2 loading control in DANs derived from 4 iPD patients and 4 healthy controls. (D) Western blots from three independent experiments (n = 3) were quantified by densitometry and P-ERK1/2 normalized to a total ERK1/2. Graphs show all individual data points. Data is shown as healthy vs iPD as well as iPD data stratified for T/T and C/C genotype. (E) Ratio of P-ERK1/2 (normalized to total ERK1/2) following CZC treatment/untreated. All error bars represent standard deviations.
(PDF)

S6 Fig Uncropped WB membranes for LRRK2 detection in lysates of SF-TAP tagged iPSCs. Related to Fig 3. Detection of (A) LRRK2, (B) LRRK2 pS935, (C) LRRK2 pS910, (D) LRRK2 pS955, (E) GAPDH, (F) b-actin. 50 μg of total protein were loaded. Membranes were probed using the indicated antibodies at the specified dilutions, and developed for the time length reported in each panel.
(PDF)

S7 Fig Uncropped WB membranes for LRRK2 detection in IP eluates of SF-TAP tagged iPSCs. Related to Fig 3. Detection of (A) LRRK2, (B) LRRK2 pS935, (C) LRRK2 pS910, (D) LRRK2 pS955. 30 μl of eluate were loaded. Membranes were probed using the indicated antibodies at the specified dilutions, and developed for the time length reported in each panel.
(PDF)

S8 Fig Uncropped WBs used for quantification. Related to Figs 5–7. Detection of (A) LC3B, (B) LAMP1, (C) ATP5A, (D) Tom20, (E) alpha-Synuclein in iPD iPSC-derived dopaminergic neurons as well as in healthy age- and gender-matched controls. Protein levels are normalized by the indicated housekeeping protein. iPD genotypes are reported. Blots show protein levels in the presence or absence of a specified treatment. Baf = bafilomycin, Rot = rotenone, Val = valinomycin, CZC = *CZC-*25146
(PDF)

S1 Table Primers used in this study. (PDF)

S2 Table Calculated variances from western blot data. (PDF)
