# Supplementary figures and images for: Generation of iPSCs carrying a common LRRK2 risk allele for in vitro modeling of idiopathic Parkinson's disease
Source: PLoS One. 2018 Mar 7;13(3):e0192497. doi: 10.1371/journal.pone.0192497 (PMC5841660; doi:10.1371/journal.pone.0192497)

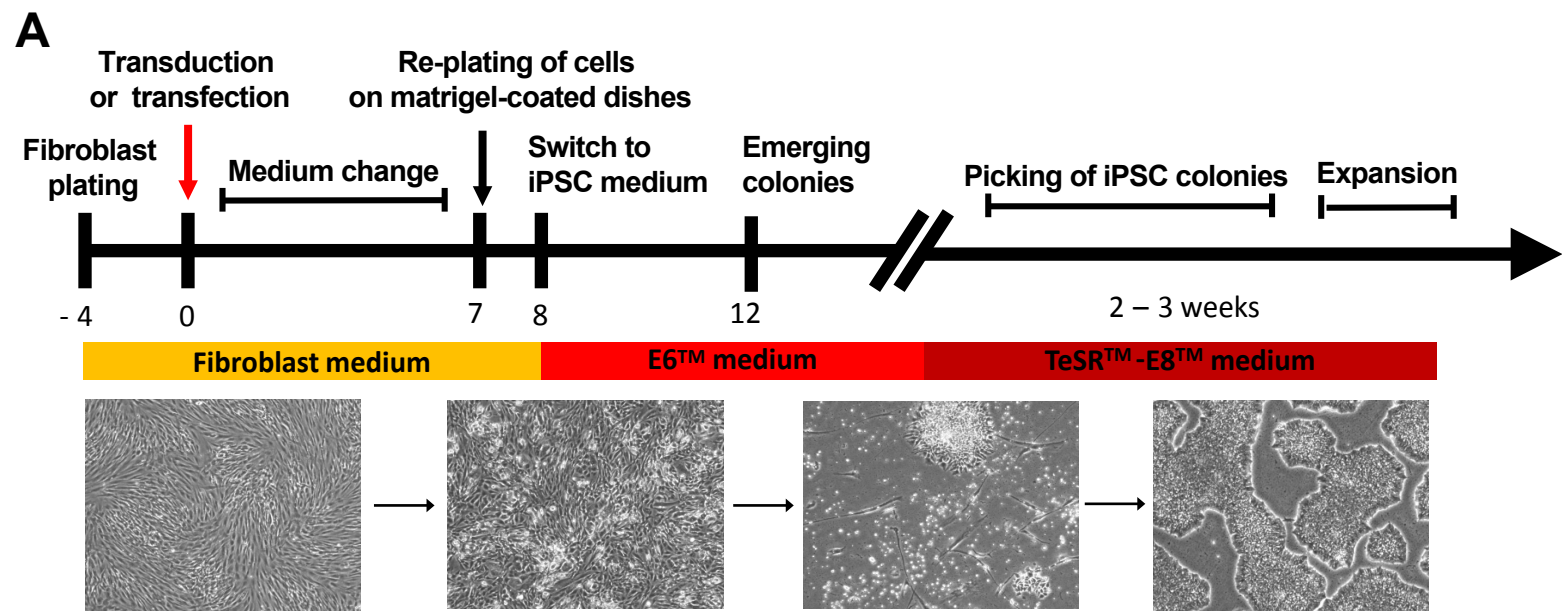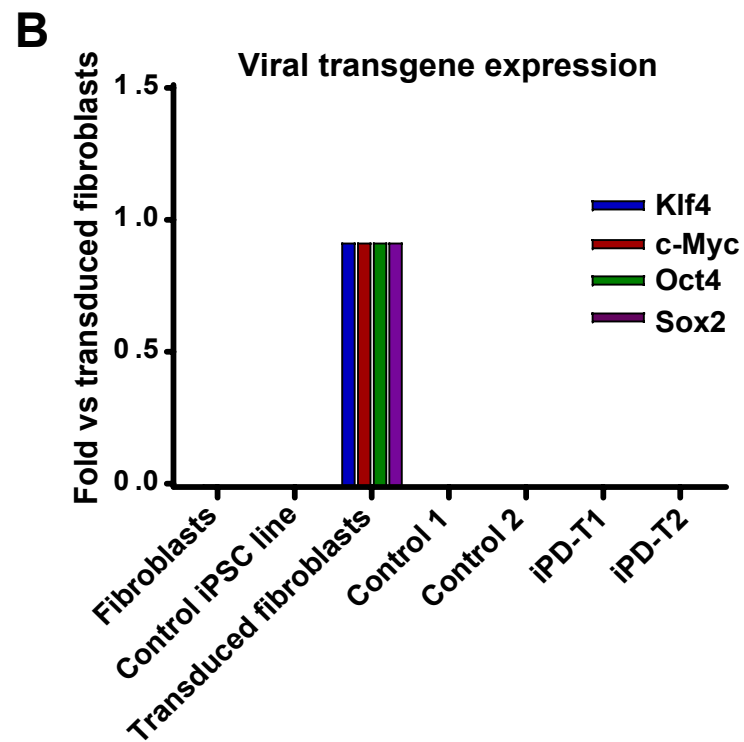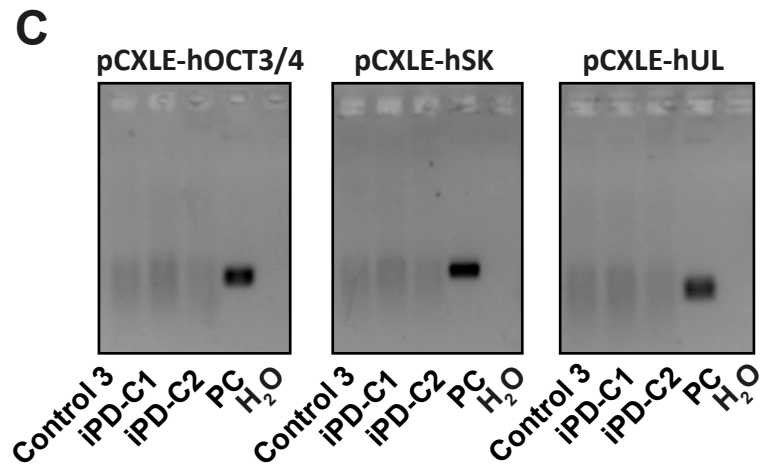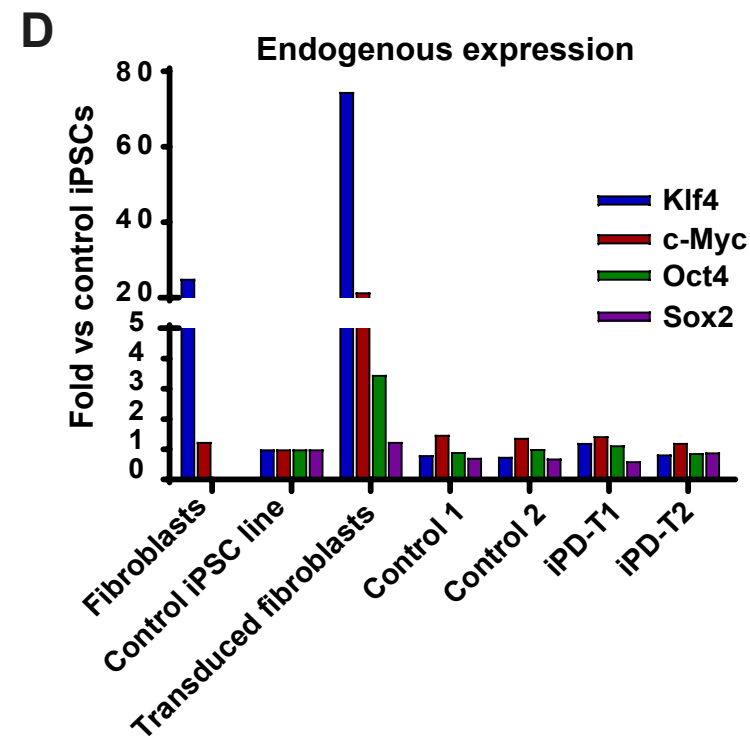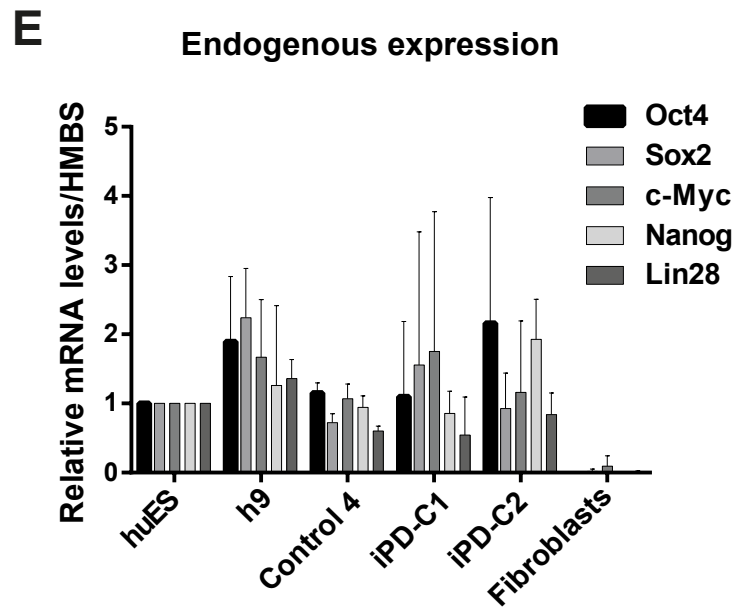

Supplement: S1 Fig — (A) Schematic representation of the reprogramming protocol used in this study. Two control and two iPD iPSC lines were generated by Sendai virus infection. The remaining control and iPD iPSC lines were obtained by plasmid transfection. (B) qRT-PCR results showing the absence of viral transgene expression in the generated iPSC lines after passage P7. (C) Agarose gel showing that lines reprogrammed via plasmid transfection do not express plasmidic pluripotency transgenes. A PCR-amplified product is only present in the positive control for the experiment. (D) qRT-PCR results showing successful induction of pluripotency marker expression in target cells after infection. Values are expressed as fold to a control iPSC line. (E) qRT-PCR results showing successful induction of pluripotency marker expression in target cells upon plasmid transfection. Values are expressed as fold to a control human embryonic stem cell line (huES). (PDF) [file pone.0192497.s001.pdf]

**DNA****PAX6****NESTIN****SOX1****Overlay****Control 1**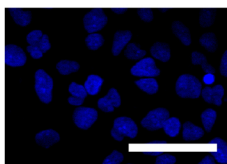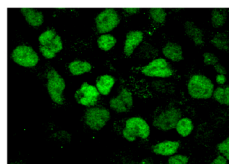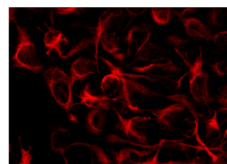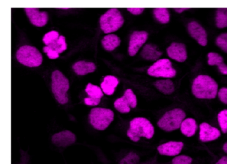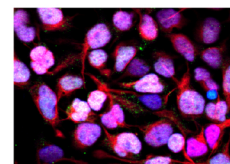**Control 2**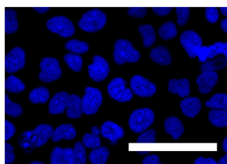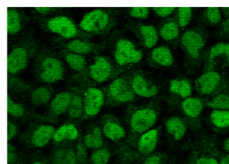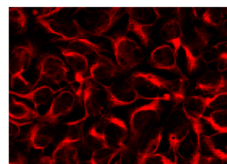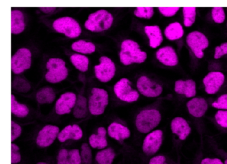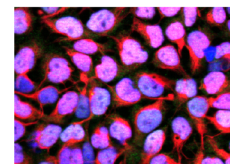**Control 3**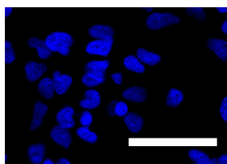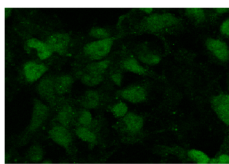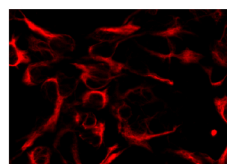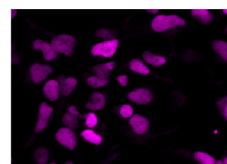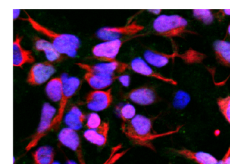**Control 4**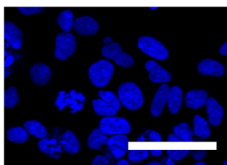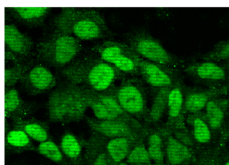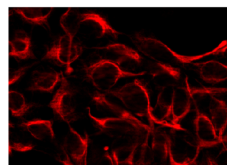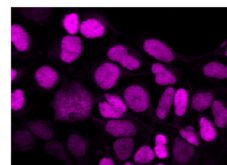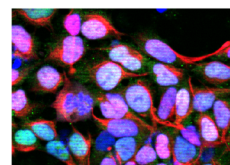**iPD-T1**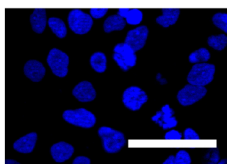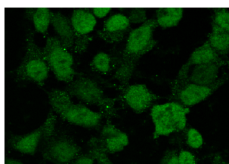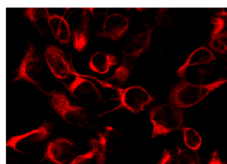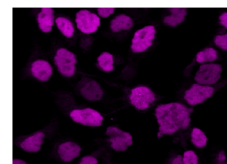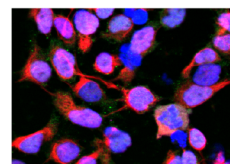**iPD-T2**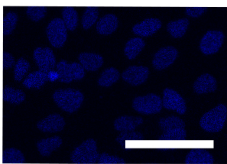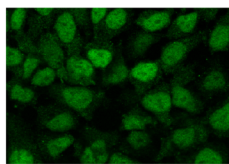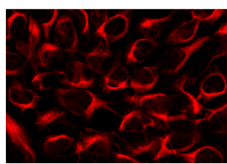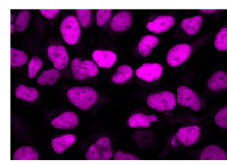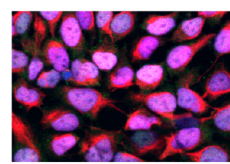**iPD-C1**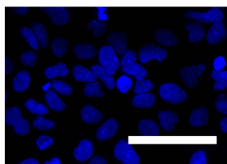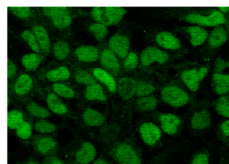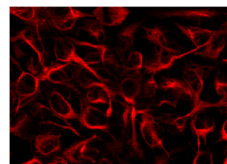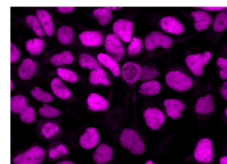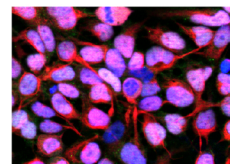**iPD-C2**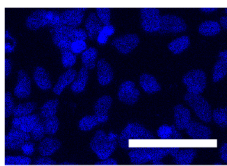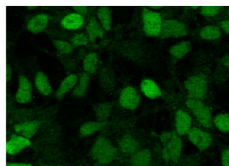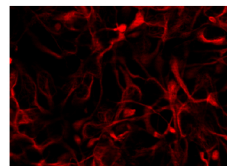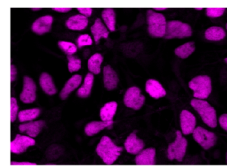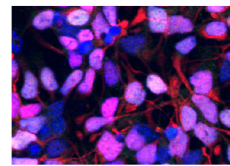

Supplement: S3 Fig — Fluorescent micrographs of iPSC-derived smNPCs for the indicated markers. Scale bar is 50 μm. (PDF) [file pone.0192497.s003.pdf]

**A**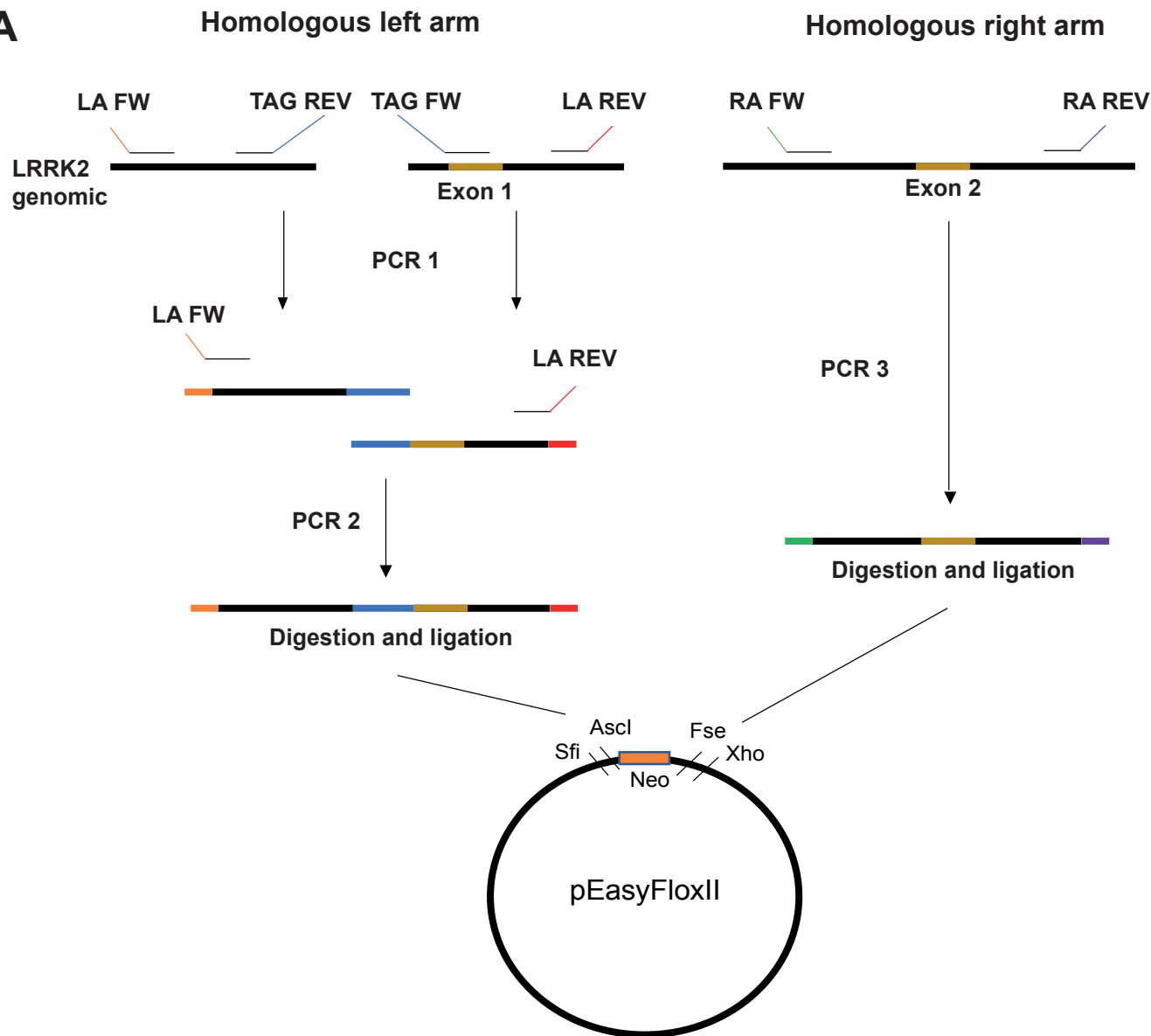**B**    **Genotyping**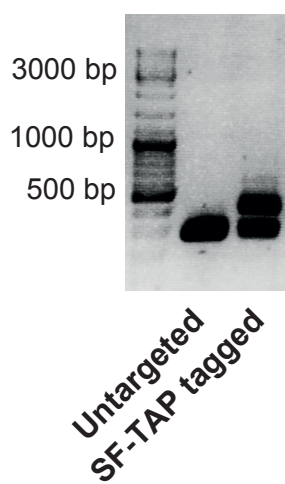**C**    **Tagged LRRK2 mRNA**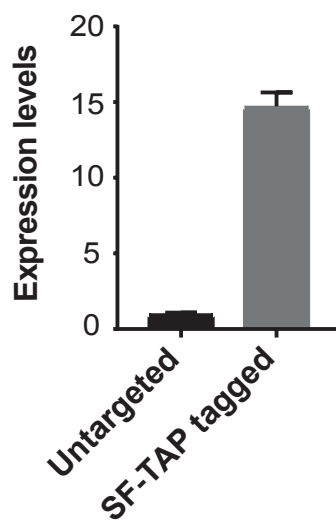**D**    **LRRK2 mRNA**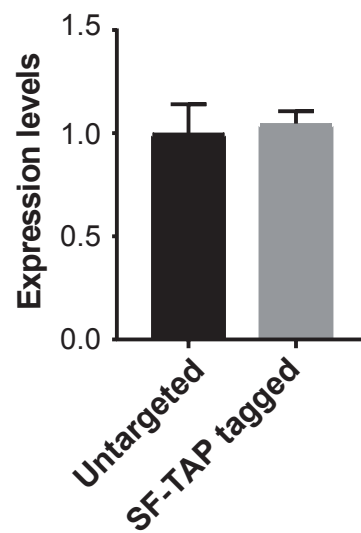**E**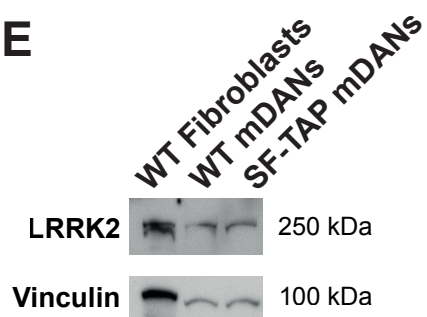**F**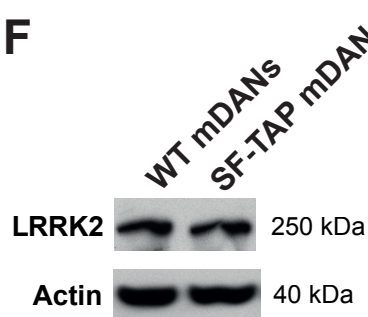

Supplement: S4 Fig — (A) Generation of the LRRK2 SF-TAP TAG donor construct. Primers containing the SF-TAP TAG sequence were designed to amplify two separate products of the homologous left arm from genomic DNA (PCR 1). An overlapping PCR from those products generated the final homologous left arm containing tagged exon 1 as well as Sfi and AscI restriction sites (orange, blue) (PCR 2). The homolgous right arm was amplified directly amplified from genomic DNA containing the restriction sites Fse and Xho (green, purple) (PCR 3). Left and right arm were then digested and ligated in the pEasyFloxII donor construct. LA = left arm, RA = right arm. (B) DNA agarose gel reveals the heterozygous integration of the SF-TAP tag in the LRRK2 locus. Primers were designed to amplify exon 1 of LRRK2 outside of the targeted region. (C) qRT-PCR analysis proving expression of tagged LRRK2 in DANs. (D) qRT-PCR analysis showing that endogenous LRRK2 expression in LRRK2 tagged and wildtype DANs is not altered. E) Western blot for LRRK2 performed in WT fibroblasts, WT DANs and SF-TAP tagged DANs. Vinculin was used as a loading control. (F) Western blot for LRRK2 performed in WT and SF TAP tagged DANs using actin as a loading control. (PDF) [file pone.0192497.s004.pdf]

**A**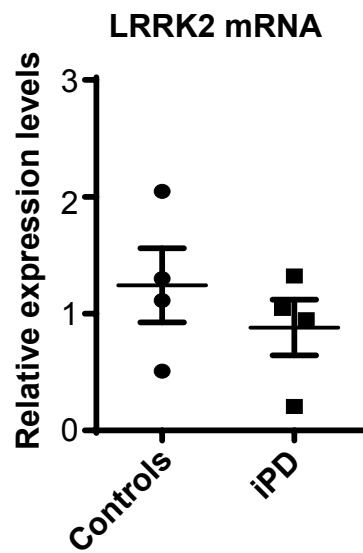**B**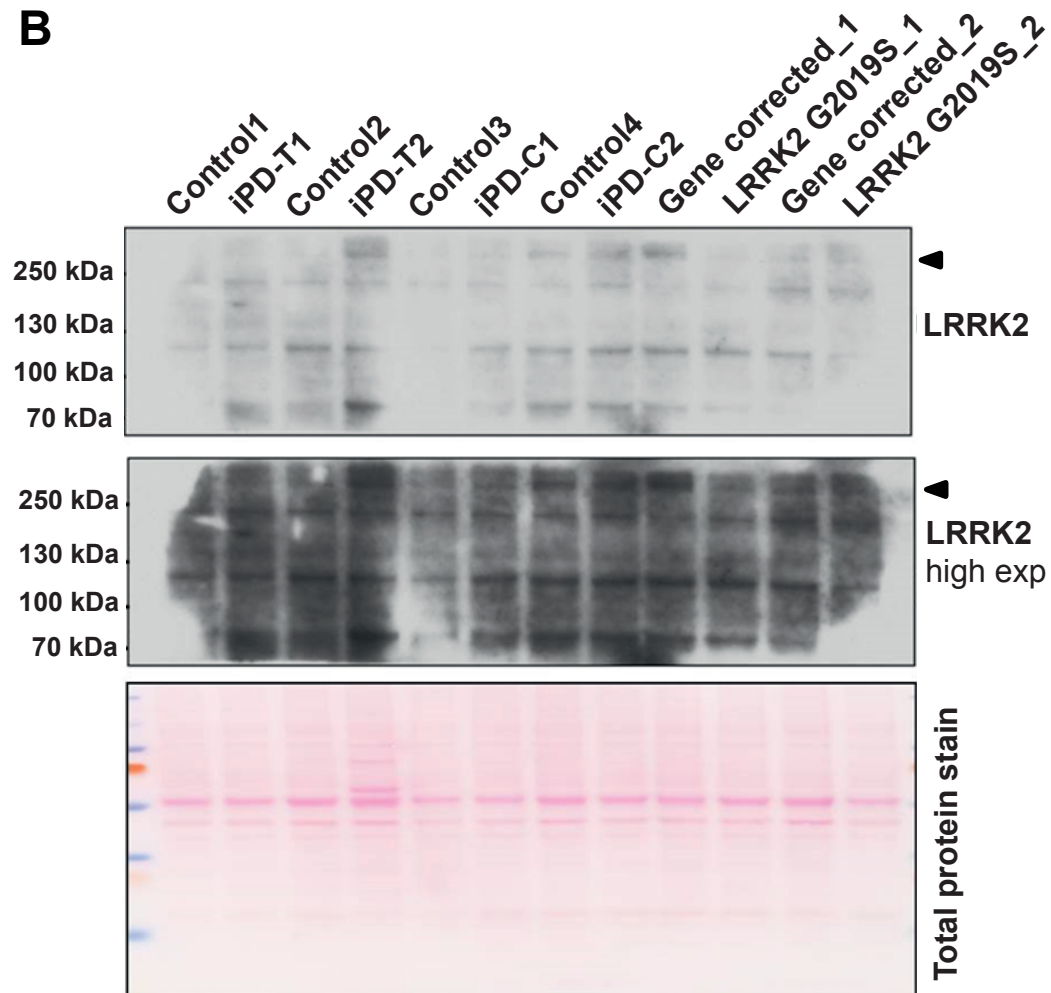**C**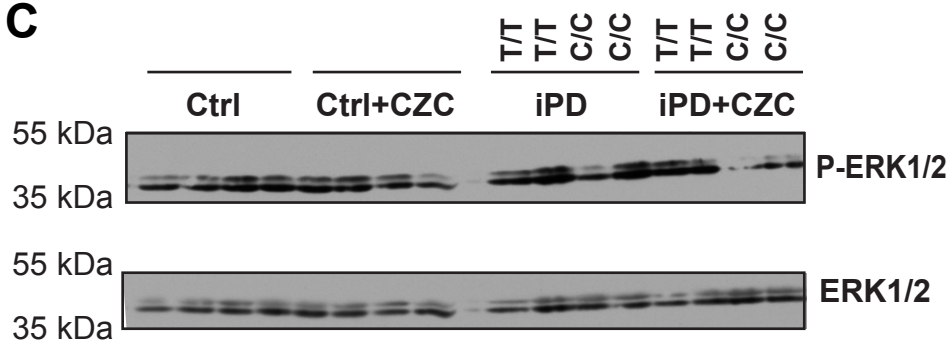**D**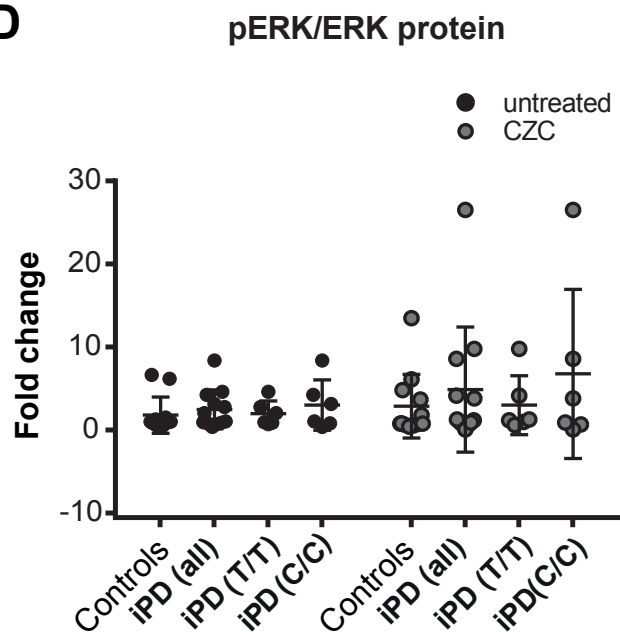**E**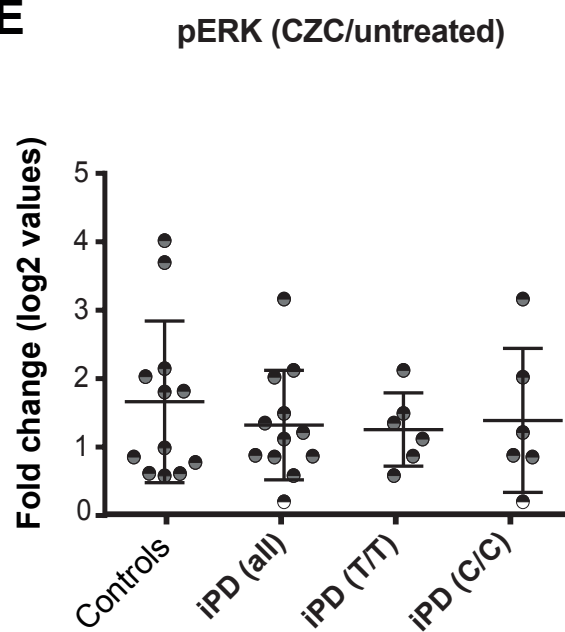

Supplement: S5 Fig — (A) qRT-PCR data showing LRRK2 mRNA expression levels in healthy controls and iPD lines. Each point represents average values of duplicates for each individual sample. No significant differences are detected between the groups. (B) Representative western blot of LRRK2 detected in iPD, age and gender-matched controls, and isogenic lines using an anti-LRRK2 antibody. Detection of endogenous LRRK2 in DANs requires protein purification and high concentrations for better resolution. (C) Representative western blot of P-ERK1/2 and total ERK1/2 loading control in DANs derived from 4 iPD patients and 4 healthy controls. (D) Western blots from three independent experiments (n = 3) were quantified by densitometry and P-ERK1/2 normalized to a total ERK1/2. Graphs show all individual data points. Data is shown as healthy vs iPD as well as iPD data stratified for T/T and C/C genotype. (E) Ratio of P-ERK1/2 (normalized to total ERK1/2) following CZC treatment/untreated. All error bars represent standard deviations. (PDF) [file pone.0192497.s005.pdf]

**A** Ab a-LRRK2 MJFF2, 1:5000, o.n., 4°C, 15 sec

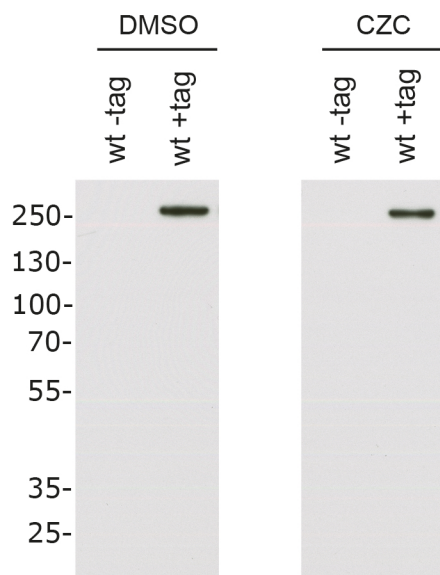

**B** Ab a-LRRK2 pS935, 1:2000, o.n., 4°C, 1 min

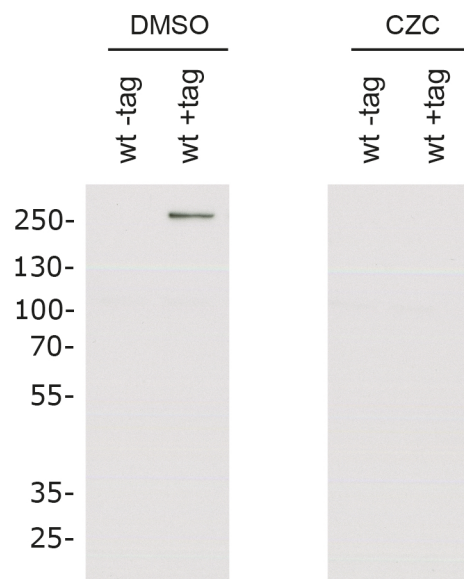

**C** Ab a-LRRK2 pS910, 1:2000, o.n., 4°C, 5 min

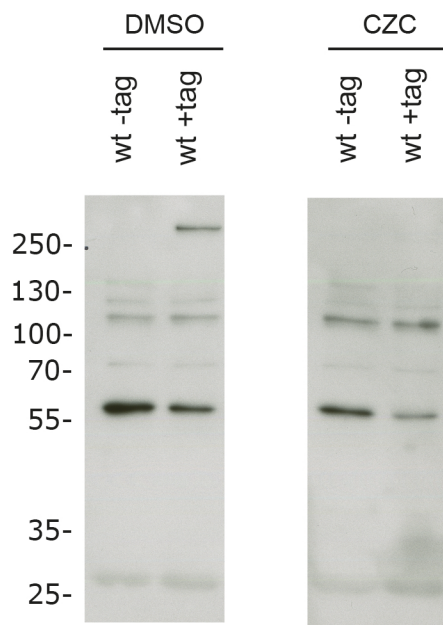

**D** Ab a-LRRK2 pS955, 1:2000, o.n., 4°C, 10 min

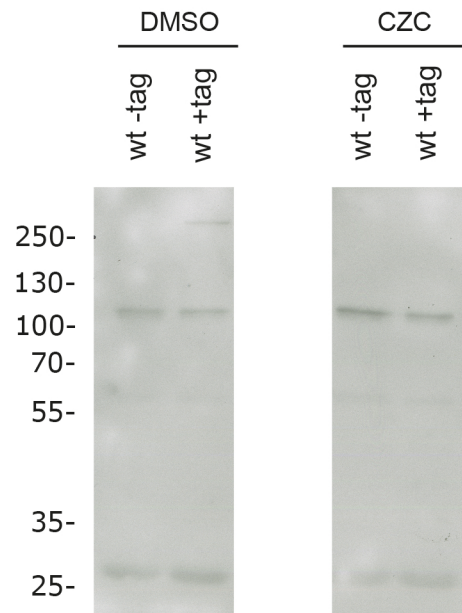

Supplement: S7 Fig — Related to Fig 3. Detection of (A) LRRK2, (B) LRRK2 pS935, (C) LRRK2 pS910, (D) LRRK2 pS955. 30 μl of eluate were loaded. Membranes were probed using the indicated antibodies at the specified dilutions, and developed for the time length reported in each panel. (PDF) [file pone.0192497.s007.pdf]

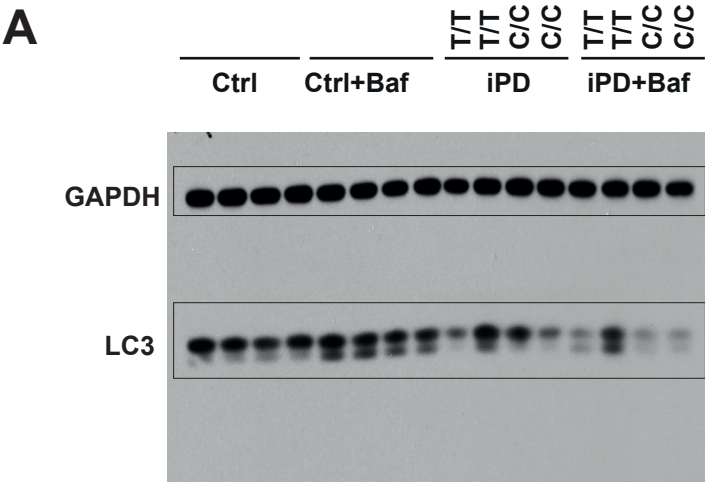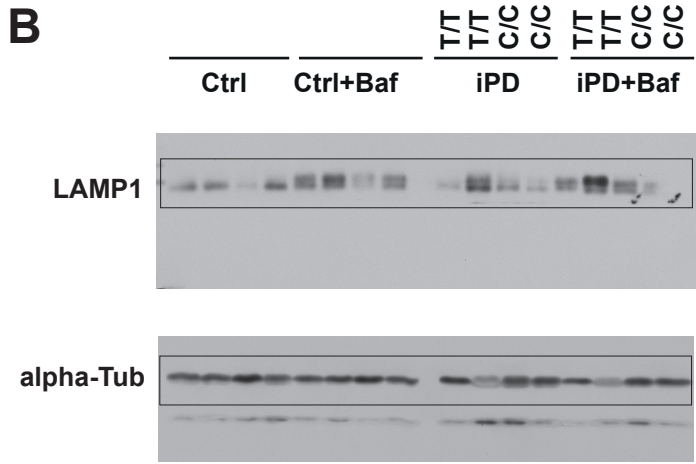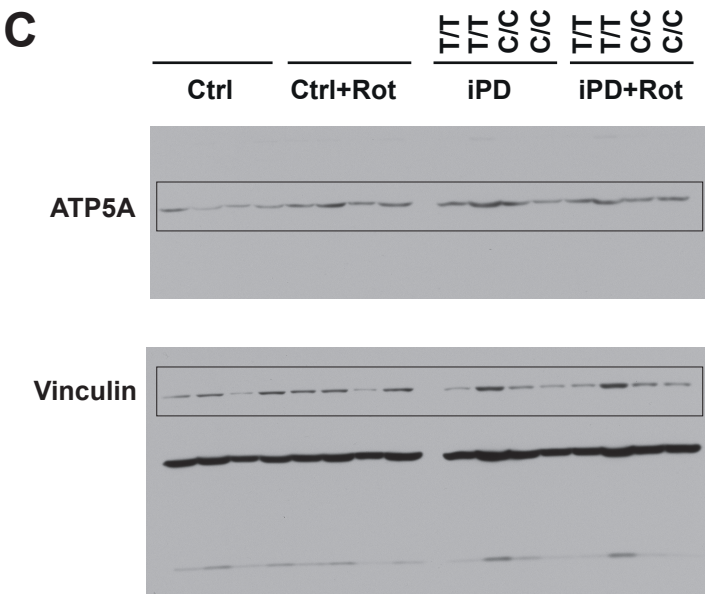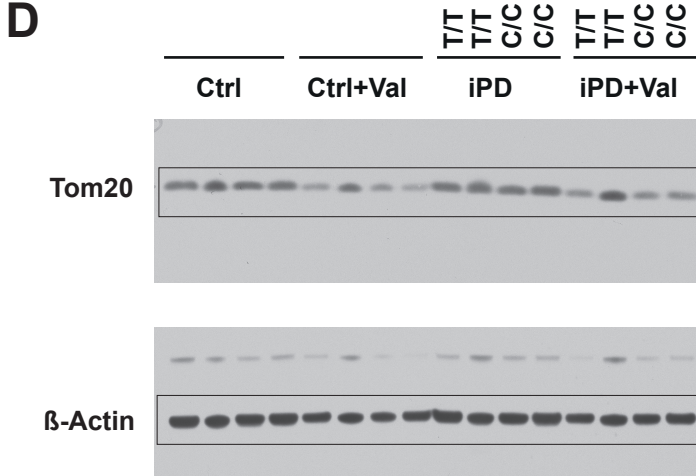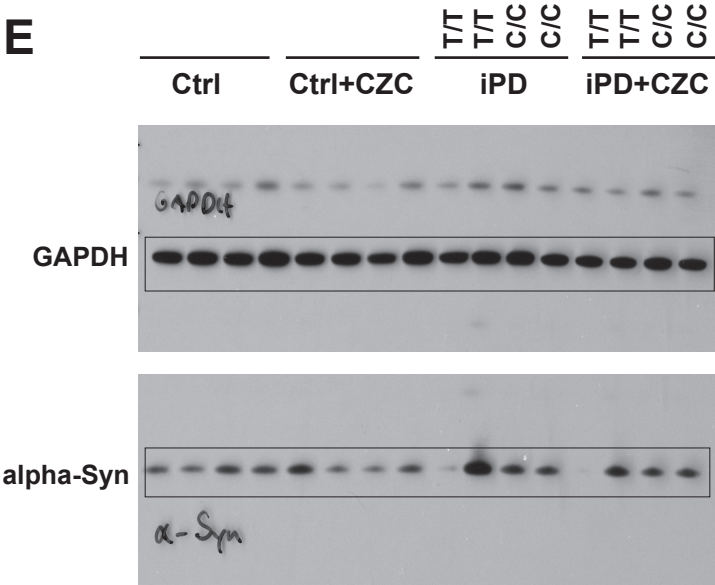

Supplement: S8 Fig — Related to Figs 5–7. Detection of (A) LC3B, (B) LAMP1, (C) ATP5A, (D) Tom20, (E) alpha-Synuclein in iPD iPSC-derived dopaminergic neurons as well as in healthy age- and gender-matched controls. Protein levels are normalized by the indicated housekeeping protein. iPD genotypes are reported. Blots show protein levels in the presence or absence of a specified treatment. Baf = bafilomycin, Rot = rotenone, Val = valinomycin, CZC = CZC-25146 (PDF) [file pone.0192497.s008.pdf]
