# Supplementary material for: Generation of iPSCs carrying a common LRRK2 risk allele for in vitro modeling of idiopathic Parkinson's disease
Source: PLoS One. 2018 Mar 7;13(3):e0192497. doi: 10.1371/journal.pone.0192497 (PMC5841660; doi:10.1371/journal.pone.0192497)
Supplement: S2 Fig — (A) Metaphase spreads and SNP microarray demonstrating euploid karyotype of the iPSCs generated in this study. Each chromosome with the corresponding B allele frequency and log R ratio is shown. (B) Quality control-paternity test results demonstrating that the genotype of each mDAN line corresponds to that of the putative parental fibroblast cell line. 11599 = Control 1; 16424 = Control 2; 19301 = iPD C1; 16423 = Control 3; 16421 = Control 4; 19390 = iPD T1; 19925 = iPD C2; 21032 = iPD T2. (PDF) [file pone.0192497.s002.pdf]

A

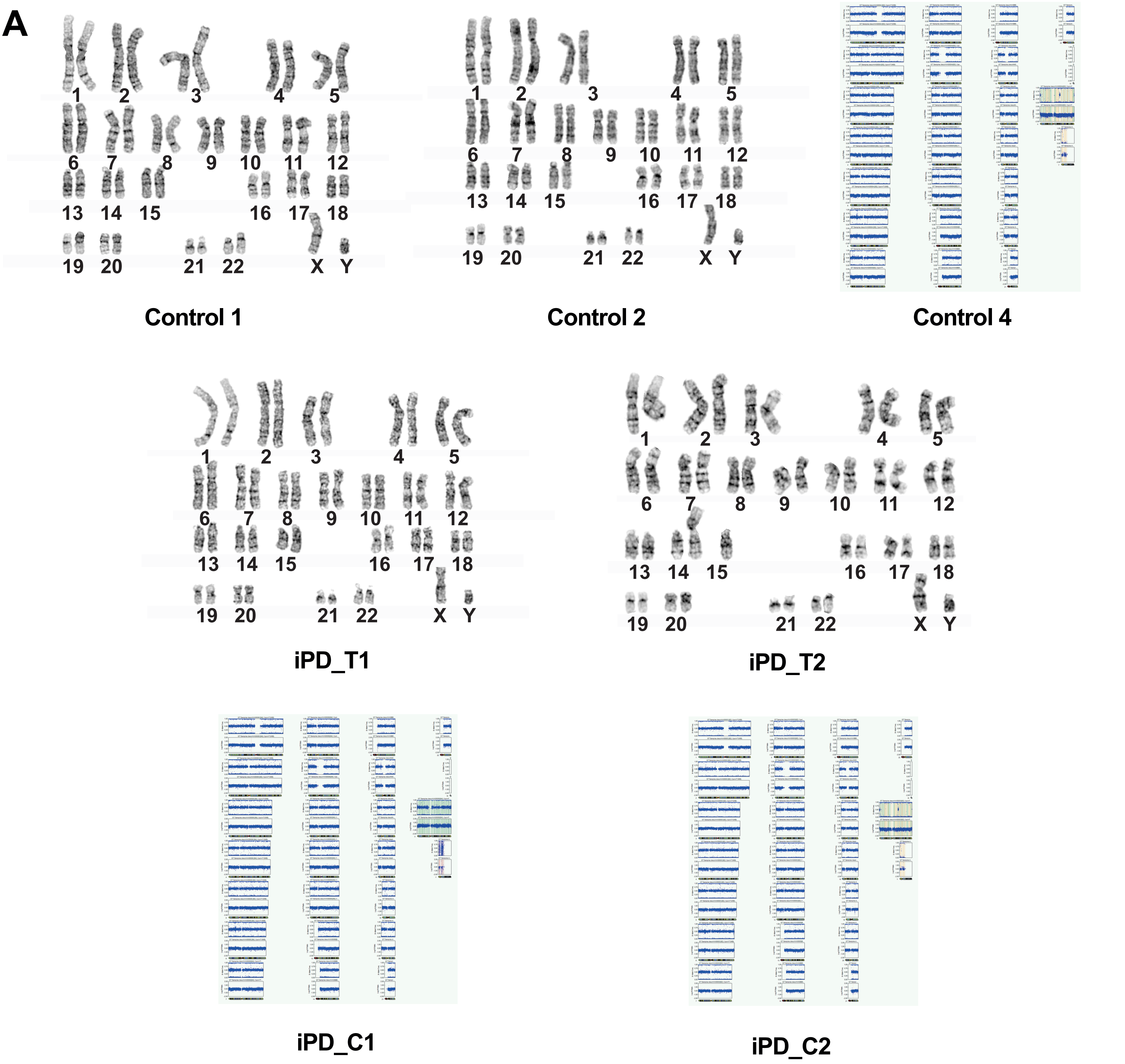

B

Sample ID	ABO	ABO-Genotype (11 SNPs)	ID-SNP-Genotyp (16 SNPs, 2 of which on Y)	Result
11599_fibroblast	A/A	G-A-C-C-T-C-G-C-G-G-C	T\C-A\G-T\C-G-G-A\T-G\T-A\G-G\A-G-A-C\T-A\G-G\A-G-A	Matching
11599_mDAN	A/A	G-A-C-C-T-C-G-C-G-G-C	T\C-A\G-T\C-G-G-A\T-G\T-A\G-G\A-G-A-C\T-A\G-G\A-G-A	
16424_fibroblast	A/0	delG\G-A\G-C-C-T\A-C-G\A-C\T-G-G-C	T-A\G-T\C-G\A-G\C-A\T-G\T-A\G-G\A-C\G-A-C-G-G\A-G-G	Matching
16424_mDAN	A/0	delG\G-A\G-C-C-T\A-C-G\A-C\T-G-G-C	T-A\G-T\C-G\A-G\C-A\T-G\T-A\G-G\A-C\G-A-C-G-G\A-G-G	
19301_fibroblast	0/0	delG-A-C-C-T-C-G-C-G-G-C	T\C-A\G-T\C-G\A-G\C-A\T-G-G-G\A-C\G-A\G-T-A-G-G-G	Matching
19301_mDAN	0/0	delG-A-C-C-T-C-G-C-G-G-C	T\C-A\G-T\C-G\A-G\C-A\T-G-G-G\A-C\G-A\G-T-A-G-G-G	
16421_fibroblast	B/0	G-G-C-G-T-C\T-G-C-G\C-G\A-C	T-A-T-G-C-A\T-T-G-G\A-C-A\G-C\T-A\G-A-G-G	Matching
16421_mDAN	B/0	G-G-C-G-T-C\T-G-C-G\C-G\A-C	T-A-T-G-C-A\T-T-G-G\A-C-A\G-C\T-A\G-A-G-G	
16423_fibroblast	0/0	delG-A\G-C-C-T\A-C-G\A-C\T-G-G-C	T\C-A\G-T\C-G-G\C-A-T-A\G-G\A-C\G-A-C-A\G-G-X-X	Matching
16423_mDAN	ND	delG-A-C-no info-T-C-G-C\T-G-G-C	T\C-A\G-T\C-G-G\C-A-T-A\G-G\A-C\G-A-C-A\G-G-X-X	
19390_fibroblast	B/0	delG\G-A\G-C-C\G-T-C\T-G-C-G\C-G\A-C	T-A-T-G\A-C-A-G\T-G-A-C\G-A\G-C-G-G\A-G-G	Matching
19390_mDAN	B/0	delG\G-A\G-C-C\G-T-C\T-G-C-G\C-G\A-C	T-A-T-G\A-C-A-G\T-G-A-C\G-A\G-C-G-G\A-G-G	
19925_fibroblast	A/0	delG\G-A\G-C\T-C-T\A-C-G\A-C\T-G-G-C\delC	T\C-G-T\C-G\A-G\C-A\T-G\T-A-G\A-C-G-C-A\G-A-X-X	Matching
19925_mDAN	A/0	delG\G-A\G-C\T-C-T\A-C-G\A-C\T-G-G-C\delC	T\C-G-T\C-G\A-G\C-A\T-G\T-A-G\A-C-G-C-A\G-A-X-X	
21032_fibroblast	A/0	delG\G-A-C-C-T-C-G-C-G-G-C	C-A-T-A-G\C-A\T-G\T-A\G-G\A-C-A-C\T-A\G-G-G-G	Matching
21032_mDAN	A/0	delG\G-A-C-C-T-C-G-C-G-G-C	C-A-T-A-G\C-A\T-G\T-A\G-G\A-C-A-C\T-A\G-G-G-G	
