# Supplementary material for: Generation of iPSCs carrying a common LRRK2 risk allele for in vitro modeling of idiopathic Parkinson's disease
Source: PLoS One. 2018 Mar 7;13(3):e0192497. doi: 10.1371/journal.pone.0192497 (PMC5841660; doi:10.1371/journal.pone.0192497)
Supplement: S6 Fig — Related to Fig 3. Detection of (A) LRRK2, (B) LRRK2 pS935, (C) LRRK2 pS910, (D) LRRK2 pS955, (E) GAPDH, (F) b-actin. 50 μg of total protein were loaded. Membranes were probed using the indicated antibodies at the specified dilutions, and developed for the time length reported in each panel. (PDF) [file pone.0192497.s006.pdf]

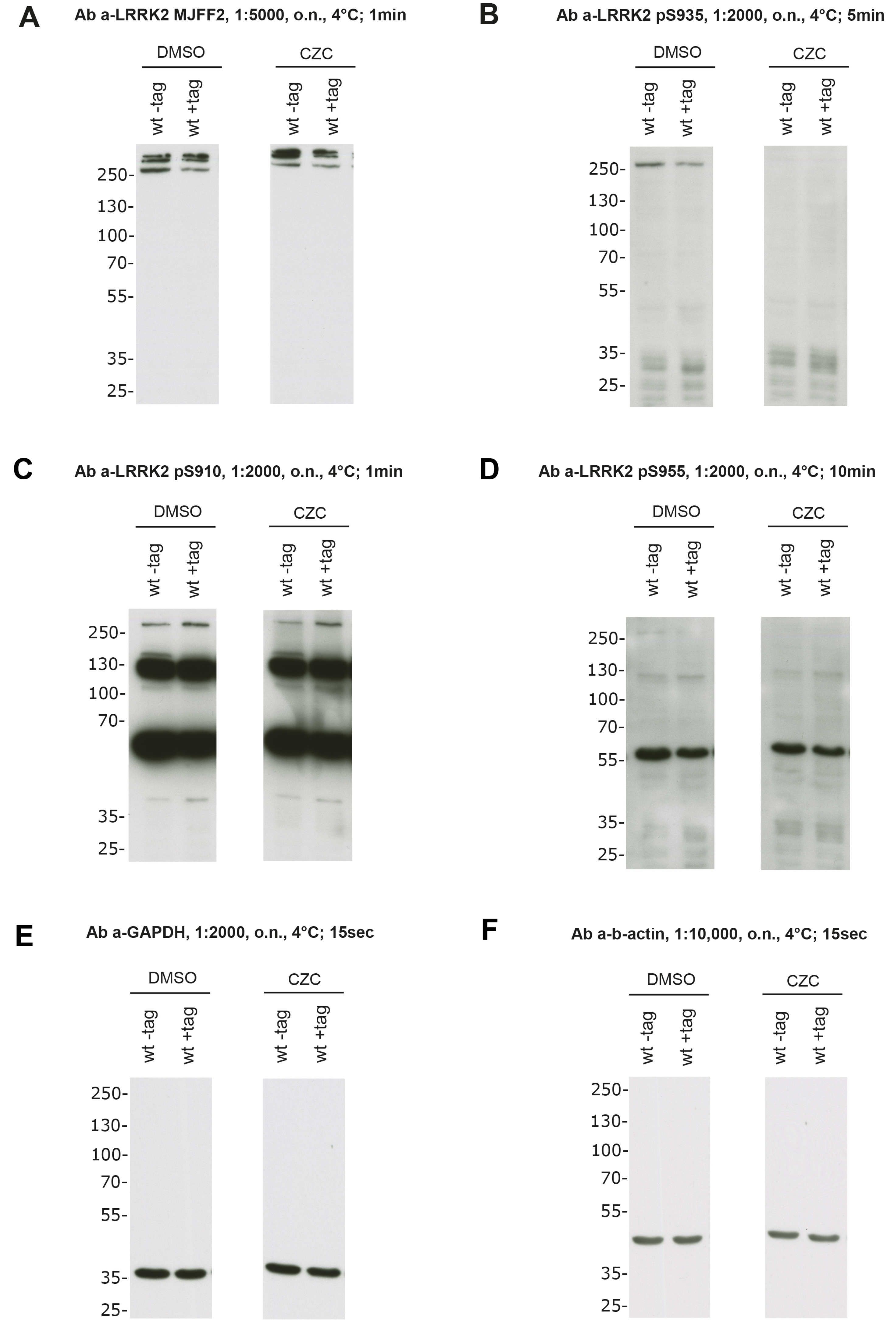
A
Ab a-LRRK2 MJFF2, 1:5000, o.n., 4°C; 1min
B
Ab a-LRRK2 pS935, 1:2000, o.n., 4°C; 5min
C
Ab a-LRRK2 pS910, 1:2000, o.n., 4°C; 1min
D
Ab a-LRRK2 pS955, 1:2000, o.n., 4°C; 10min
E
Ab a-GAPDH, 1:2000, o.n., 4°C; 15sec
F
Ab a-b-actin, 1:10,000, o.n., 4°C; 15sec
DMSO
CZC
wt -tag
wt +tag
250-
130-
100-
70-
55-
35-
25-
