# Supplementary material for: Generation of iPSCs carrying a common LRRK2 risk allele for in vitro modeling of idiopathic Parkinson's disease
Source: PLoS One. 2018 Mar 7;13(3):e0192497. doi: 10.1371/journal.pone.0192497 (PMC5841660; doi:10.1371/journal.pone.0192497)
Supplement: S1 Table — (PDF) [file pone.0192497.s009.pdf]

Gene	Forward primer (5'-3')	Reverse primer (3'-5')
GAPDH	CTGGTAAAGTGGATATTGTTGCCAT	TGGAATCATATTGGAACATGTAAACC
endo OCT4	GGAAGGAATTGGGAACACAAAGG	AACTTCACCTTCCCTCCAACCA
endo SOX2	TGGCGAACCATCTCTGTGGT	CCAACGGTGTCAACCTGCAT
endo KLF4	ACAGTCTGTTATGCACTGTGGTTTCA	CATTTGTTCTGCTTAAGGCATACTTGG
endo c-MYC	CCAGCAGCGACTCTGAGGA	GAGCCTGCCTCTTTTCCACAG
NANOG	CCTGTATTTGTGGGCCTG	GACAGTCTCCGTGTGAGGCAT
LIN28	GGAGGCCAAGAAAGGGAATATGA	AACAATCTTGTGGCCACTTTGACA
viral KLF4	TTCCTGCATGCCAGAGGAGCCC	AATGTATCGAAGGTGCTCAA
viral c-MYC	TAACTGACTAGCAGGCTTGTCG	TCCACATACAGTCCTGGATGATGATG
viral KOS	ATGCACCGCTACGACGTGAGCGC	ACCTTGACAATCCTGATGTGG
viral SeV	GGATCACTAGGTGATATCGAGC	ACCAGACAAGAGTTTAAGAGATATGTAT
MAPT	CTCGCATGGTCAGTAAAAGCAA	GGGTTTTTGCTGGAATCCTGGT
LRRK_1	TGGGTTGGTCACTTCTGTGC	CATTGGCTGGAAATGAGTGC
LRRK2_2	CCGAGCAGCCTATGAAGGAG	ACCCTACAGCACAGGATTGC
LRRK2 tag	GATGATGACGACAAATCCGC	GGTCACTTCTGTGCAAATTAA
aSYN	GGAGTGGCCATTCGACGAC	CCTGCTGCTTCTGCCACAC
