# Supplementary material for: Generation of iPSCs carrying a common LRRK2 risk allele for in vitro modeling of idiopathic Parkinson's disease
Source: PLoS One. 2018 Mar 7;13(3):e0192497. doi: 10.1371/journal.pone.0192497 (PMC5841660; doi:10.1371/journal.pone.0192497)
Supplement: S2 Table — (PDF) [file pone.0192497.s010.pdf]

LC3B

Groups	*Count*	*Sum*	*Average*	*Variance*
WT	16	3,582281	0,223893	0,025904
IPD	16	4,826194	0,301637	0,035826
TT	8	2,017913	0,252239	0,017777
CC	8	2,808281	0,351035	0,036212

LAMP1

Groups	*Count*	*Sum*	*Average*	*Variance*
WT	12	2,673019	0,222752	0,039669
IPD	12	3,375396	0,281283	0,051492
TT	12	4,843598	0,403633	0,020298
CC	12	1,402123	0,116844	0,006044

ATP5A

Groups	*Count*	*Sum*	*Average*	*Variance*
WT	12	5,028775	0,419065	0,1255
IPD	12	5,000599	0,416717	0,082732
TT	12	3,087835	0,25732	0,100722
CC	12	1,760175	0,146681	0,057436

TOMM20

Groups	*Count*	*Sum*	*Average*	*Variance*
WT	16	3,131891	0,195743	0,028031
IPD	16	5,165215	0,322826	0,082645
TT	8	1,98602	0,248253	0,048598
CC	8	2,854551	0,356819	0,140787

aSYN

Groups	*Count*	*Sum*	*Average*	*Variance*
WT	12	0,979047	0,081587	0,008365
IPD	12	1,708196	0,14235	0,011861
TT	6	0,935349	0,155891	0,012947
CC	6	0,772847	0,128808	0,012432
